# An AI‐Driven Framework for Directed Self‐Assembly Toward Design‐Technology Co‐Optimization

**DOI:** 10.1002/advs.78401

**Published:** 2026-10-22

**Authors:** Caiwei Shang, Yanting Xu, Yue Zhang, Yan Zhang, Jianfeng Li, Ming Li, Longlin Lei, Wenda Bao, Weixuan Zeng, Shisheng Xiong

**Affiliations:** ^1^ College of Future Information Technology Fudan University Shanghai China; ^2^ Zhangjiang Laboratory Shanghai China; ^3^ The State Key Laboratory of Molecular Engineering of Polymers The Research Center of AI For Polymer Science Department of Macromolecular Science Fudan University Shanghai China

**Keywords:** autonomous optimization, block copolymers, design‐technology co‐optimization, directed self‐assembly, intelligent manufacturing, inverse design, Ohta‐Kawasaki modeling

## Abstract

Directed self‐assembly (DSA) provides a promising route for sub‐10‐nm patterning, but its integration into design‐technology co‐optimization (DTCO) remains limited by the difficulty of translating self‐assembly process constraints into manufacturable layout solutions. Here, an artificial‐intelligence‐driven closed‐loop simulation framework for DSA is presented to convert experimentally constrained DSA process knowledge into a process‐constrained design‐space map for DSA‐aware layout decomposition. The framework integrates a three‐dimensional Ohta‐Kawasaki model with Bayesian optimization and convolutional neural network screening, enabling automated model calibration using experimental racetrack‐template data without manual tuning. It efficiently identifies optimal model parameters, achieving a mean simulation‐experiment error below 1.5 nm while maintaining morphological consistency. In addition, four multimodal large language models are further evaluated for DSA morphology screening and are shown to adapt to new image modalities and defect criteria without additional task‐specific fine‐tuning, relying solely on text‐based rules and few‐shot examples. The calibrated model enables target‐constrained inverse design of experimentally validated template windows, translating DSA constraints into a manufacturable solution‐space map for layout decomposition. Its extension to peanut‐shaped templates without additional parameter calibration further supports DSA‐aware layout decomposition and DTCO. Overall, this work establishes an intelligent closed‐loop workflow linking experiment, simulation, and design, providing a practical route toward DTCO.

## Graphical abstract

An artificial intelligence (AI)‐driven closed‐loop framework integrates experiment, three‐dimensional physics‐based simulation, and template design for directed self‐assembly (DSA). Guided by experimental data, AI‐assisted calibration accelerates model optimization, supports morphology prediction, and identifies template windows. Transfer across racetrack and peanut‐shaped geometries demonstrates model reusability. The workflow converts DSA process constraints into manufacturable design spaces for layout decomposition and design‐technology co‐optimization.

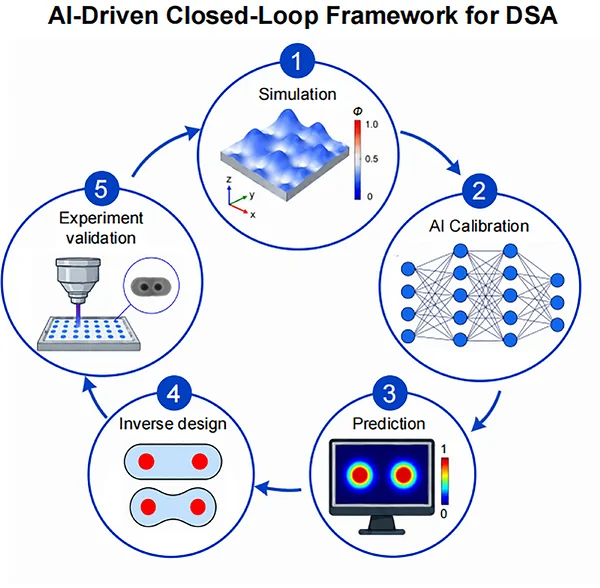

## 1 Introduction

As logic technology nodes enter the sub‐10‐nm regime, conventional 193‐nm immersion lithography faces increasing limitations from resolution constraints and process complexity. Design rules at critical layers have become increasingly stringent, and advanced static random access memory (SRAM) fabrication requires tighter control over pattern fidelity and placement accuracy [1]. Although extreme ultraviolet (EUV) lithography with a numerical aperture (NA) of 0.33 is widely used for high‐volume manufacturing at advanced logic nodes, further scaling at the tightest pitches may require EUV double patterning, increasing process complexity, cost, and overlay requirements [2]. The introduction of 0.55‐NA high‐NA EUV can improve the resolution achievable with a single exposure but requires substantial investment in new exposure tools, masks, materials, and metrology infrastructure [3]. This transition also places greater demands on the electronic design automation (EDA) ecosystem, including lithography‐aware design, layout decomposition, mask optimization, and design‐technology co‐optimization (DTCO) [4]. In this context, directed self‐assembly (DSA) of block copolymers (BCPs) has emerged as a promising complementary patterning strategy, enabling periodic nanostructures to form through molecular self‐organization at relatively low cost. By generating multiple nanoscale features from a relatively sparse lithographic guiding pattern, DSA provides intrinsic feature‐size scaling and pattern multiplication capabilities [5], with the potential to reduce the number of masks, exposure steps, and associated process complexity [6]. These advantages make DSA particularly attractive for interconnect and via patterning [7]. Among its applications, hole multiplication is regarded as a promising route toward near‐term manufacturable deployment and is therefore the focus of this work.

However, the scalable deployment of DSA for via patterning faces several challenges, and a framework linking design intent with process feasibility remains underdeveloped. First, defectivity control and morphological stability are highly sensitive to the process window. Even small changes in guiding‐template geometry or surface affinity can induce pattern distortion or defect formation [8]. Second, process‐parameter optimization often relies on repeated experimental iterations [9]. In practice, each validation requires the full process flow, including template fabrication, BCP film coating, annealing, structural characterization and pattern transfer. These laborious cycles render systematic exploration of high‐dimensional parameter spaces inefficient, increasing development cost and limiting throughput. To support mechanistic studies and process assessment, several simulation approaches have been developed, including self‐consistent field theory (SCFT), Monte Carlo (MC) methods and dissipative particle dynamics (DPD) [10, 11, 12, 13, 14, 15]. Although these methods provide rigorous physical descriptions, their high computational cost limits the effective coupling between simulation outputs and large‐scale experimental datasets. This limitation becomes more pronounced in artificial‐intelligence‐driven, data‐intensive research, where model training, parameter optimization and high‐throughput data generation require rapidly increasing computational resources. Consequently, large‐scale iterative evaluations based on high‐fidelity physical models remain impractical, which restricts systematic model calibration and scalable predictive deployment. On the other hand, simulations without experimental benchmarking may fail to capture realistic process conditions, compromising predictive reliability. Some studies have combined computationally efficient compact models with experimental measurements to improve predictive accuracy, but such alignment often depends on manual parameter tuning or empirical fitting, typically using no more than five experimental datasets [16, 17, 18]. This strategy is therefore poorly suited to calibration against larger datasets and remains labor‐intensive, time‐consuming and prone to convergence difficulties when multiple parameters are coupled. Recently, Cao et al. developed a Bayesian calibration framework with machine‐learning surrogate acceleration to infer Ohta‐Kawasaki (OK) model parameters for unguided BCP self‐assembly [19]. For practical template‐guided DSA, calibration must further account for consistency across multiple template conditions and morphology validity, introducing additional requirements for process‐oriented model calibration. Third, as device dimensions continue to shrink and design complexity increases, conventional integrated‐circuit (IC) layout design rules do not explicitly incorporate the physics of BCP self‐assembly or the constraints imposed by guiding templates. Introducing DSA into existing design rules can therefore lead to template‐BCP mismatch, defect accumulation, narrowed process windows and degraded manufacturability. Consequently, layout decomposition and design‐for‐manufacturability (DFM) place increasingly stringent demands on DTCO, highlighting the need for a systematic DSA‐based framework that integrates design, simulation and process constraints. Existing studies have integrated the DSA process into lithography and layout design from multiple perspectives. Early efforts primarily focused on lithography‐DSA co‐optimization, using forward simulation and coupled optimization to evaluate DSA behavior under prescribed design conditions [20, 21, 22, 23]. While valuable for process coordination, these approaches are inherently forward‐looking and offer limited means to derive manufacturable design spaces from target requirements. Subsequent studies established inverse DSA by recovering guiding templates from prescribed target morphologies [24, 25, 26, 27, 28]. These methods typically yield a single deterministic template for a given target, rather than constructing a multi‐solution feasible design space that captures practical process tolerance; consequently, they cannot rapidly supply the multiple manufacturable template candidates required by layout decomposition and iterative design flows. Inverse DSA was further extended to inverse lithography, in which the guide pattern and mask are sequentially optimized to connect target contact patterns with lithographic patterning [29]. However, these workflows remain predominantly simulation‐driven and lack systematic calibration or closed‐loop refinement, which constrains their reliability in practical process optimization. A systematic comparison of these approaches is provided in Table S1.

Beyond the immediate DSA context, closed‐loop approaches integrating computational modeling, experimental feedback, and iterative decision‐making are increasingly being explored for materials discovery and optimization [30]. Such approaches offer a general strategy for navigating coupled design spaces through iterative model‐evaluation feedback. To address the DSA‐specific challenges outlined above, we propose an AI‐driven closed‐loop simulation framework for DSA‐enabled logic patterning, as schematically illustrated in Figure 1a. The framework is designed to convert experimentally constrained DSA process knowledge into a process‐constrained design‐space map for DSA‐aware layout decomposition, and comprises a forward calibration/prediction stage and an inverse‐design stage constrained by the target center‐to‐center distance (CCD). Unlike data‐driven inverse‐design approaches that directly learn the mapping from target DSA patterns to guiding templates [31], the present framework retains a physics‐based model as the basis for prediction and inverse design, with AI serving as an enabling layer for experimental calibration and automated model refinement.

**FIGURE 1:**
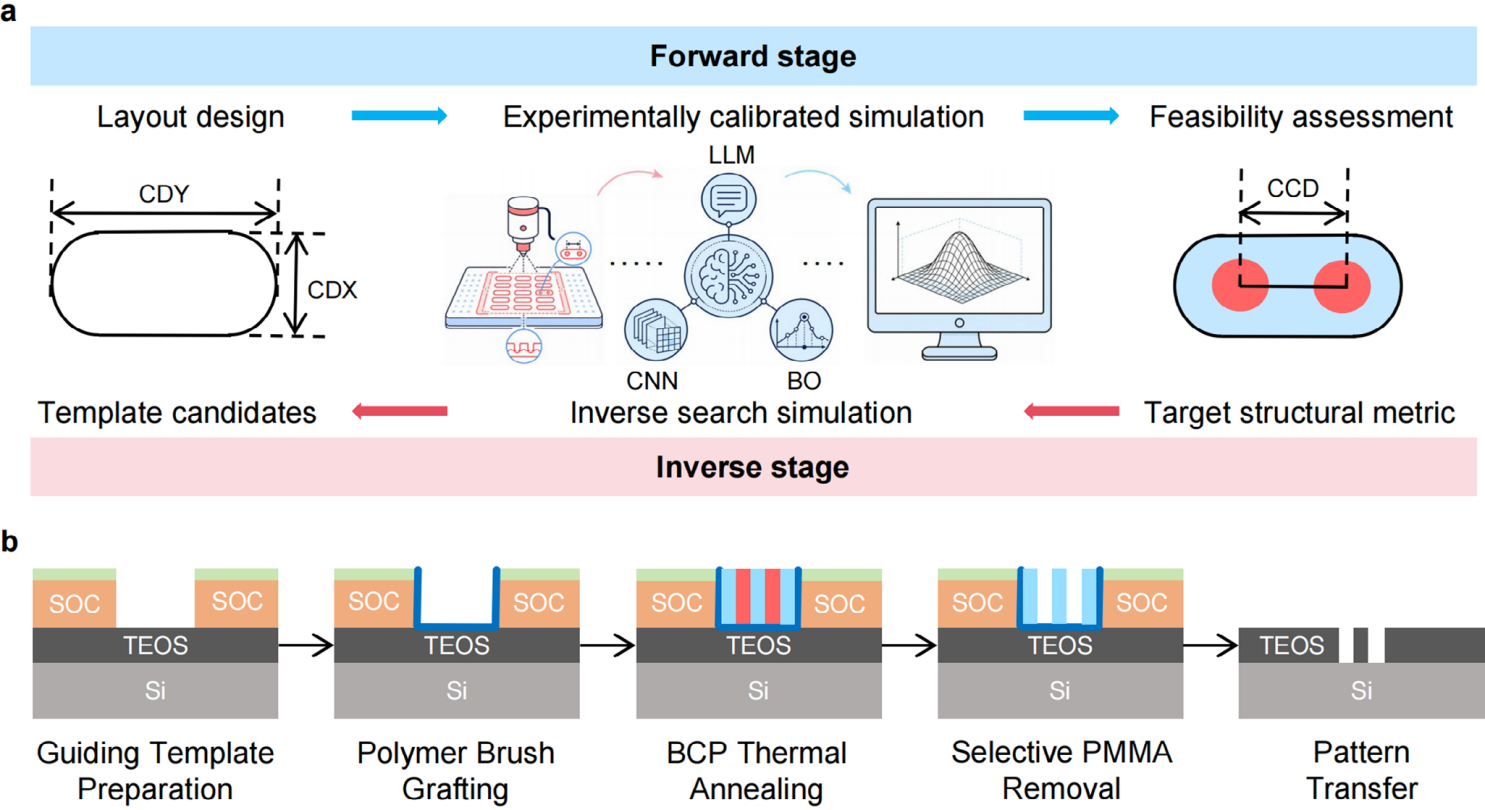
Integrated DSA closed‐loop framework and experimental workflow for hole multiplication. (a) Schematic of the DSA closed‐loop simulation framework integrating forward prediction and inverse design through an experimentally calibrated simulation model. (b) Schematic illustration of the experimental procedure for DSA hole multiplication. SOC, spin‐on carbon; TEOS, tetraethyl orthosilicate; PMMA, poly(methyl methacrylate).

In the forward stage, layout design serves as the starting point for constructing and parameterizing guiding‐template geometries. Using experimental data from racetrack‐template‐based DSA hole multiplication, we establish an automated calibration and evaluation framework that integrates Bayesian optimization (BO) for parameter learning with a convolutional neural network (CNN) for morphology assessment [32, 33, 34, 35]. CNN‐based defect recognition has been widely used in semiconductor manufacturing for automated defect detection and classification from wafer and scanning electron microscopy (SEM) images [36, 37, 38]. In contrast, the CNN in our work operates on simulation‐derived DSA composition‐density fields and serves as a morphology‐validity classifier within the BO calibration loop. This framework enables adaptive optimization of a three‐dimensional (3D) compact OK model and experimentally aligned prediction of structural outcomes, while substantially reducing the need for manual evaluation of parameter feasibility and thereby supporting pre‐experimental assessment of template feasibility. To reduce the dependence of CNN‐based classification on re‐annotation and retraining when extended to new image formats, defect criteria, and morphology types, multimodal vision‐language models offer an alternative pathway via instruction following and in‐context learning. Visual instruction tuning enables general vision‐language understanding, while a handful of image‐text demonstrations can support few‐shot adaptation without additional task‐specific fine‐tuning [39, 40]. Recent studies have further demonstrated the potential of such models for zero‐shot classification of scientific microscopy images [41]. Given this cross‐scenario adaptability, we evaluate four multimodal large language models (LLMs) for DSA morphology classification across different viewing modalities using text‐defined criteria and few‐shot reference images. Within the present workflow, these models function as complementary screening tools for scenarios involving new image formats or evaluation criteria, whereas the CNN remains the primary classifier that feeds the optimization loop. The target CCD, a key structural metric in hole multiplication, characterizes the achievable spacing under a given intrinsic material period and therefore serves as a direct measure of patterning capability. We then impose CCD as a design constraint and use the calibrated model to inversely identify multiple template geometries within the DSA‐accessible process window that satisfy the target CCD. Because the CCD of a given template must remain within the DSA‐accessible process window to ensure stable pattern generation, the identified template solutions can be propagated back to the design stage, thereby providing multiple manufacturable options for process‐constrained, DSA‐aware layout decomposition [42, 43]. Through this bidirectional closed‐loop workflow, experimental trial‐and‐error is reduced, and a manufacturable multi‐solution design space for layout decomposition is established, thereby improving overall iteration efficiency.

Distinct from conventional single‐pass and unidirectional optimization strategies, the proposed AI‐driven closed‐loop simulation framework establishes an efficient, dynamic and bidirectional feedback workflow. The same calibrated model is further applied to peanut‐shaped templates without additional parameter calibration to assess cross‐geometry predictive capability. By linking experiment, simulation and layout design, this framework provides a practical foundation for DSA‐aware layout decomposition and DTCO in advanced logic patterning.

## 2 Simulation Model and Methods

### 2.1 Ohta‐Kawasaki Model

The proposed framework is based on the traditional OK model [44]. The bulk free energy of the diblock copolymer, F, is expressed as

(1) $$\begin{array}{ccc} F\;\left[\Psi \left(r\right)\right] & = & c_{2}\;\Psi {\left(r\right)}^{2}+c_{4}\Psi {\left(r\right)}^{4}+c_{5}{\left[\nabla \Psi \left(r\right)\right]}^{2}\\ & & +\;c_{7}\int dr^{\prime }G\left(r,r^{\prime }\right)\left[\Psi \left(r\right)-\bar{\Psi }\right]\left[\Psi \left(r^{\prime }\right)-\bar{\Psi }\right] \end{array}$$

where the order parameter, Ψ(***r***), represents the difference between the volume fractions of the minor, polymethyl methacrylate (PMMA), and major, polystyrene (PS), blocks, and $\bar{\Psi }$ denotes the spatially averaged order parameter. *G*(***r***, ***r′***) is the Green's function. The coefficients *c*_2_, *c*_4_, *c*_5_ and *c*_7_ are chosen to yield stable cylindrical morphologies in the bulk phase [17].

The surface interaction between the diblock copolymer and the chemically patterned guiding template, $F_{s}$, is described as

(2) $$\begin{array}{c} F_{s}\;\left[\Psi \left(r\right)\right]=\int dr\left\{\Lambda_{s}\left(r\right){\left[\phi_{A}\left(r\right)-\phi_{s}\right]}^{2}+\Lambda_{b}\left(r\right){\left[\phi_{A}\left(r\right)-\phi_{b}\right]}^{2}\right\} \end{array}$$

where ϕ_*A*_(***r***) is the PMMA volume fraction, whereas ϕ_*s*_ and ϕ_*b*_ denote the preferred PMMA volume fractions near the sidewalls and bottom surface, respectively. Compared with the previously reported formulation [17], this formulation increases the number of surface parameters from two to four by independently describing the sidewall and bottom interactions, enabling more flexible parameter optimization and improved agreement with experimental observations. In this study, both ϕ_*s*_ and ϕ_*b*_ are smaller than 0.5, corresponding to PS‐attractive surfaces consistent with the experimental template affinities. The coupling parameters Λ_*s*_(***r***) and Λ_*b*_(***r***) define the spatially dependent strengths of preferential interactions, with larger values indicating stronger affinities. When Λ(***r***)  =  0, the corresponding region is non‐preferential. Additional model details are provided in the Supporting Information.

### 2.2 Experimental Procedure

The experimental procedure for DSA hole multiplication is illustrated in Figure 1b. First, racetrack‐shaped guiding templates were fabricated by 193‐nm immersion lithography using an NXT 1950i scanner. A PS‐*r*‐PMMA random copolymer brush with a PS content of approximately 72% was then spin‐coated onto the templates and thermally annealed to promote grafting. The samples were subsequently rinsed with propylene glycol monomethyl ether acetate (PGMEA) to remove ungrafted chains, thereby forming a PS‐preferential background surface. A PS‐*b*‐PMMA BCP film with a cylindrical period of *L*_0_ ≈ 42.5 nm and a PMMA volume fraction of *f*  =  0.28 was then spin‐coated and thermally annealed to induce self‐assembly and form PMMA double‐cylinder domains. The PMMA domains were selectively removed by etching, and the resulting hole‐multiplication patterns were characterized to evaluate the process window and CCD. Finally, the patterns were transferred into the tetraethyl orthosilicate (TEOS) layer by dry etching to achieve stable hole‐multiplication pattern transfer. Additional experimental details are provided in the Supporting Information.

### 2.3 Automated Parameter Optimization Workflow

To achieve quantitative agreement between simulations and experiments, we develop an automated parameter‐optimization framework that couples BO with CNN‐based feedback screening, as illustrated in Figure 2a. The framework treats the template‐BCP interaction parameters, ϕ_*s*_, ϕ_*b*_, Λ_*s*_(***r***) and Λ_*b*_(***r***), in Equation (2) as optimization variables. It employs a coordinated strategy that combines adaptive parameter search with morphology‐based classification to identify parameter sets that minimize the simulation‐experiment discrepancy while preserving physical interpretability.

**FIGURE 2:**
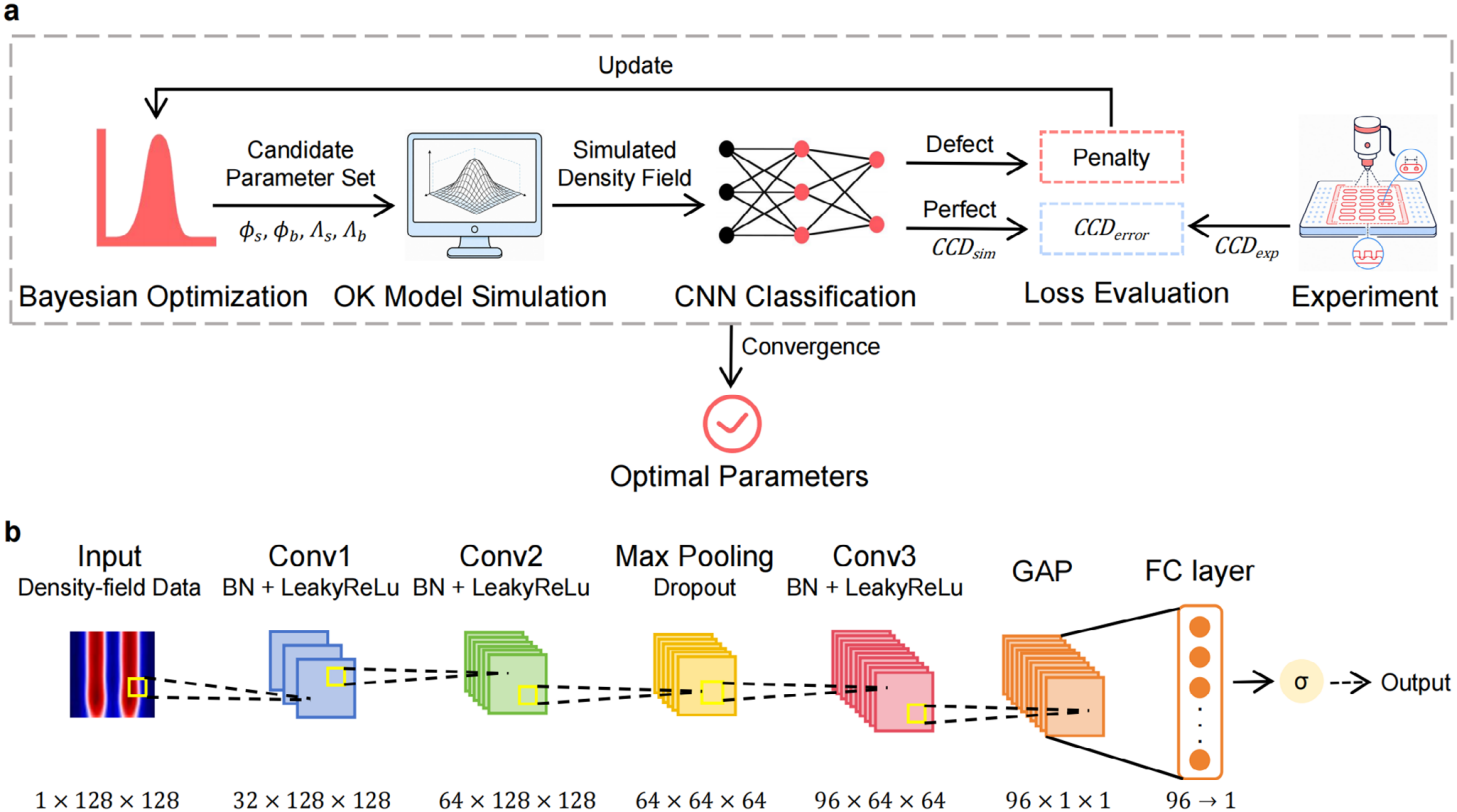
Automated BO‐CNN coupled parameter‐optimization framework and morphology classification. (a) Closed‐loop optimization workflow integrating BO with CNN feedback. (b) Baseline three‐layer CNN architecture for defect classification. Conv, convolutional layer; BN, batch normalization; LeakyReLU, leaky rectified linear unit; GAP, global average pooling; FC, fully connected layer; p, dropout probability; σ, sigmoid activation function.

In the first stage, BO is used to perform a global search within a prior‐constrained parameter space. Compared with grid‐based scans or empirically guided tuning, BO integrates statistical learning with uncertainty quantification and is well suited to simulation problems involving expensive evaluations, non‐convex objectives and inaccessible gradients. It therefore enables efficient parameter search through iterative updates based on accumulated evaluations [33, 45]. Specifically, the objective function is defined by the simulation‐experiment CCD mismatch for morphologically valid samples, whereas defective or non‐converged samples are assigned a fixed penalty and included in surrogate fitting to avoid invalid regions [46]. We adopt a Random Forest (RF) surrogate rather than the more conventional Gaussian Process because the calibration landscape exhibits non‐smooth, class‐discontinuous behavior, a regime in which RF's nonparametric tree ensemble makes fewer assumptions about the functional form and does not require kernel stationarity. The ensemble mean and the across‐tree prediction variance serve as the mean and uncertainty estimates for the expected‐improvement acquisition function. As a result, parameter calibration is transformed from an experience‐driven procedure into a systematic, data‐driven workflow with explicit uncertainty quantification.

In the second stage, CNN‐based morphology classification is used to automatically identify perfect simulation outcomes. In the high‐dimensional parameter space, a substantial fraction of simulations produce defective morphologies, such as bridging and dislocation defects. If these defective morphologies are evaluated solely through CCD errors, they may introduce misleading quantitative feedback because their apparent CCD values no longer correspond to physically valid hole‐multiplication states. Therefore, automated morphology recognition is required before simulation results are used for parameter optimization. Figure 2b illustrates the CNN architecture, which takes normalized two‐dimensional (2D) cross‐sectional composition‐density fields of size 128 × 128 as input and performs binary classification to determine whether the self‐assembled morphology is perfect. The network uses multiple convolutional layers to extract morphological features from local to global scales, together with batch normalization (BN) and LeakyReLU activation functions to improve representation capacity and training stability. Intermediate layers incorporate downsampling and regularization to mitigate overfitting while preserving key structural information. Finally, global feature aggregation followed by a lightweight classification head outputs the class probability, enabling automated determination of the morphology state. Through weight sharing and local receptive fields, the network maps high‐dimensional, spatially distributed composition‐density fields into a compact latent feature space, enabling effective extraction of discriminative features associated with hole integrity, structural connectivity and interfacial morphology. These features provide reliable morphology‐level feedback for subsequent parameter optimization. Figure S5a shows that the baseline network with three convolutional layers achieves accuracies of 84.70% and 84.85% on the training and validation sets, respectively. To further improve classification performance, we increased the network depth to four convolutional layers while preserving the overall architectural design. The resulting model achieves accuracies of 95.76%, 94.96%, and 96.56% on the training, validation, and test sets, respectively (Figure S5b,c).

However, CNN misclassification can affect subsequent BO sampling in two distinct error channels. Misclassifying a defective morphology as perfect may introduce an invalid observation into the surrogate and misdirect the search toward an infeasible region, whereas misclassifying a perfect morphology as defective may unnecessarily penalize a feasible region and reduce search efficiency. The former is more critical in the present workflow because feasible low‐error parameter solutions are non‐unique, whereas accepting a defective sample may directly bias surrogate updating. Accordingly, class‐weighted binary cross‐entropy was used to emphasize defect recognition. Confusion‐matrix analysis indicates strong discriminative capability for defective morphologies (Figure S5c), with no defective samples in the independent test set misclassified as perfect, thereby supporting reliable automated morphology screening in the BO‐CNN framework.

In the third stage, BO and CNN feedback are coupled for iterative model updating. In each BO iteration, candidate template‐BCP interaction parameters are sampled, and simulations are performed under different template conditions. The resulting morphologies are then classified by the CNN. For morphologically valid samples, the simulation‐experiment CCD error is used as the objective value. For defective or non‐converged samples, the CCD error is not evaluated; instead, a fixed large penalty is assigned. Both types of parameter‐objective pairs are included in the RF training dataset, allowing morphology‐invalid regions to be explicitly fed back to BO and discouraging subsequent sampling in these regions. This strategy progressively guides BO sampling toward stable parameter regions that are physically meaningful and experimentally relevant, reduces the need for manual feasibility checks and enables a more automated optimization workflow. Among all valid parameter sets, the set that yields the minimum overall error while maintaining a balanced error distribution across templates is selected as the optimal parameter set.

This closed‐loop workflow follows an active‐learning scheme, in which adaptive sampling is continuously refined through morphology‐based screening and feedback updates. It therefore enables efficient use of simulation data and provides reliable predictive guidance. Further details of the BO algorithm and CNN architecture are provided in the Supporting Information.

### 2.4 Multimodal LLM‐Based Morphology Screening as a Complement to CNN‐Based Screening

To improve the adaptability of morphology assessment across different image modalities and evaluation criteria, we further evaluated application programming interface (API)‐based multimodal LLMs as a complementary assessment tool for the existing CNN classifier. Whereas CNNs typically require additional sampling, annotation and retraining when the template morphology, viewing perspective, image modality or defect type changes, multimodal LLMs can be applied to new assessment tasks without additional task‐specific training [39, 40, 41]. By combining image inputs, text‐defined criteria and a few reference examples, LLMs generate structured classification outputs, enabling rapid deployment and cross‐scenario transfer for DSA morphology screening.

To avoid model‐specific conclusions, four multimodal LLMs, namely Qwen3.6‐Plus (Alibaba Cloud), GLM‐5V‐Turbo (Z.AI), GPT‐5.4 (OpenAI), and Gemini 3.1 Pro Preview (Google), were evaluated in parallel [47, 48, 49, 50]. The influence of prompt design on morphology classification was further examined using three hierarchical prompt settings. Prompt A served as the zero‐shot baseline and directly classified the test image. Prompt B incorporated global morphology criteria, whereas Prompt C further introduced few‐shot reference examples. Specifically, C‐2, C‐4 and C‐8 corresponded to one perfect and one defect reference image, two perfect and two defect reference images, and four perfect and four defect reference images, respectively, each accompanied by descriptive annotations. Each prompt setting was repeated five times, and all LLMs were required to return classification results in a strict JavaScript Object Notation (JSON) format to enable automated parsing and statistical analysis. Model performance was evaluated using accuracy, recall_defect_ and recall_perfect_, together with system‐level metrics including label‐reason consistency, API cost and average inference time. The trained CNN classifier was evaluated on the same test set as a supervised‐learning baseline. Details of the dataset split, prompt templates and structured output examples are provided in the Supporting Information.

To comprehensively compare classification performance and computational efficiency across different model‐prompt combinations, we calculated a weighted score based on key performance and system‐level metrics. The individual metric results and scoring rules are provided in the Supporting Information.

As shown in Figure 3a, the side‐view grayscale dataset was first used as a binary classification test case. Among the four models, Gemini 3.1 Pro Preview achieves the highest overall score of 81.5, with only limited variation across different prompt settings, indicating good classification stability. In contrast, the other models are more sensitive to prompt design, although the introduction of more explicit morphology criteria and reference examples improves the performance of some models. Compared with the CNN baseline score of 97.0, the overall performance of the LLMs remains lower, suggesting that the subtle grayscale variations and through‐thickness perturbations in side‐view images require more specialized feature discrimination. Nevertheless, the recall_defect_ results show that the LLMs maintain relatively stable defect‐recognition capability (Figure S11), supporting their use as preliminary screening tools for defect rejection. The representative side‐view misclassification cases show that false rejection mainly occurs in samples that are manually labeled as perfect but exhibit non‐ideal vertical morphology, suggesting that stricter prompt criteria may lead to conservative misclassification. In contrast, false acceptance is mainly associated with subtle defect features, such as residual PMMA near the bottom region, which are not sufficiently emphasized in the prompt descriptions.

**FIGURE 3:**
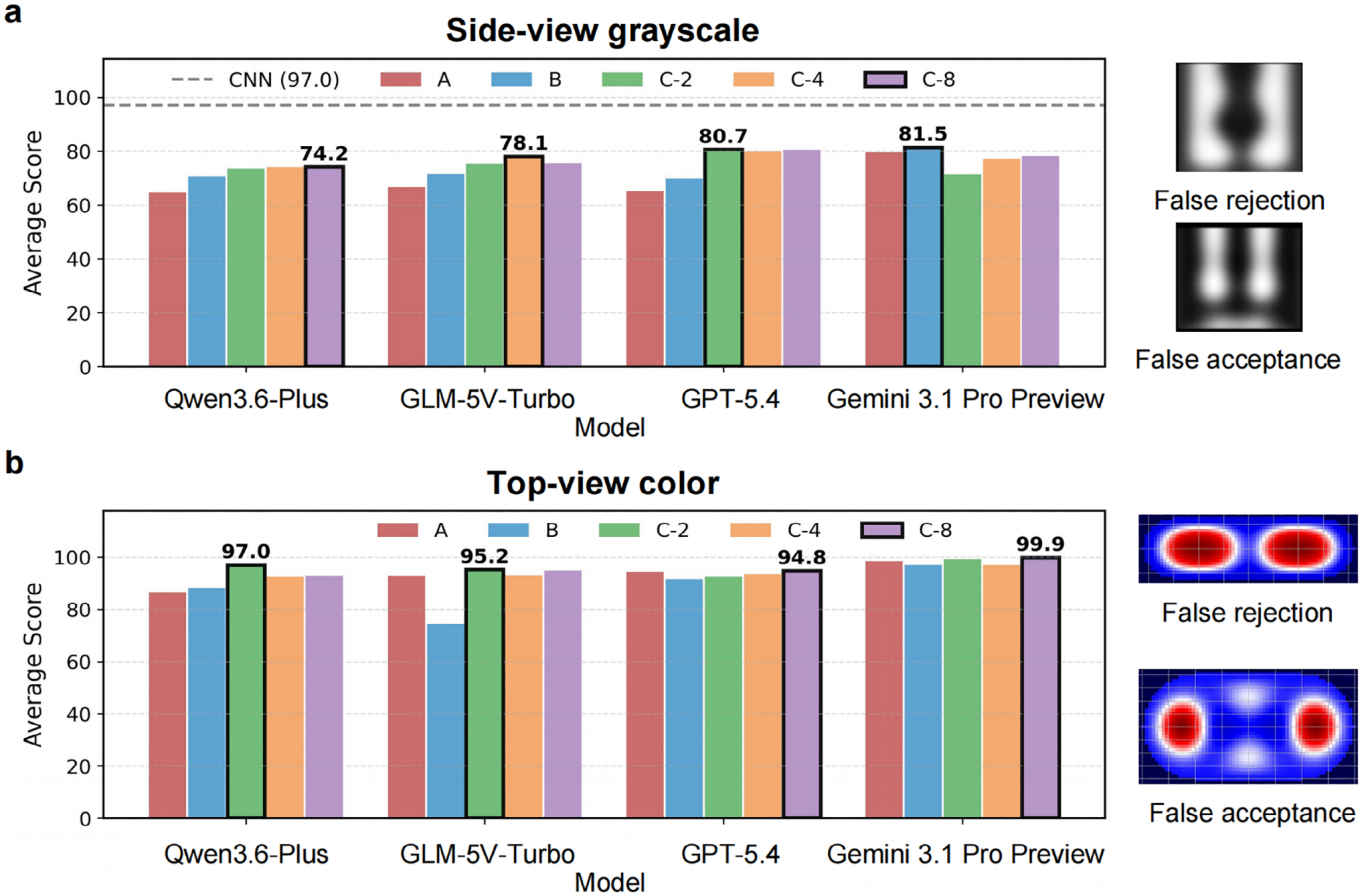
Weighted performance comparison and representative misclassification cases for LLM‐based DSA morphology screening across viewing modalities. (a) Average scores of different LLMs under side‐view grayscale inputs, with the CNN baseline indicated by dashed lines, together with representative misclassified morphologies. (b) Average scores of different LLMs under top‐view color inputs, together with representative misclassified morphologies.

Considering that top‐view morphologies are more readily accessible in practical characterization and are more suitable for data accumulation and experimental validation, we further extended the classification task to top‐view color images to assess the adaptability of LLMs across different image modalities and viewing perspectives. As shown in Figure 3b, the overall scores of all four LLMs increase markedly in this task, with Gemini 3.1 Pro Preview reaching the highest score of 99.9. Compared with side‐view grayscale images, top‐view color images enhance the visual contrast among hole structures, background regions, and defect areas through color mapping, making topological defects such as bridging, extra holes, missing holes, and incorrect hole counts easier to identify. In addition, the classification criteria for top‐view images are mainly based on geometric features, including circular integrity, spatial separation, and hole count, which are more consistent with the shape‐recognition and spatial‐relation reasoning capabilities of general vision models. As a result, the LLMs exhibit higher classification accuracy and stability in the top‐view color task. For top‐view misclassification cases, false rejection is primarily related to locally imperfect circularity, whereas false acceptance tends to occur in weak‐defect or early‐stage defect cases, where small emerging PMMA domains in the central region are not recognized by the model.

These results indicate that LLM misclassifications mainly arise from the inherent subjectivity of manual labeling, incomplete morphology criteria, and subtle defect features. Further refinements to labeling criteria and prompt design are therefore expected to enhance the reliability of LLM‐based morphology screening.

Overall, the trained CNN provides higher classification accuracy and inference efficiency within a fixed training distribution, making it suitable for fine‐grained morphology discrimination. In contrast, LLMs offer greater adaptability to changes in image modality, viewing perspective, and evaluation criteria without additional task‐specific fine‐tuning. These results highlight their complementary strengths in DSA morphology screening.

## 3 Results and Discussion

### 3.1 Optimization Results, Experimental Validation and Prediction

All optimizations were performed under a unified simulation setup to ensure numerical stability and comparability across different template geometries, with *L*_0_ =  4.8 *R_g_*, a grid spacing of 0.2 *R_g_* and 1.6 × 10^5^ time steps for each simulation run.

We assembled 28 racetrack‐template experimental cases and divided them into a training set of 14 cases for parameter calibration, a validation set of 10 cases for independent assessment and a prediction set of 4 cases for prospective evaluation. Figure 4a,b compare the BO trajectories without and with CNN‐based morphology screening, respectively, highlighting differences in convergence efficiency and error stability. For BO without CNN filtering (Figure 4a), even with an evaluation budget of 100 simulations, the optimization fails to reduce the error below 1 nm. The training‐set mean error remains high, reaching nearly 7 nm at its maximum and staying above 3 nm even at its minimum. This behavior is attributable to the frequent sampling of parameter sets that produce defective morphologies, which introduce strong, non‐smooth perturbations into the objective landscape. Consequently, the error trajectory exhibits pronounced fluctuations and intermittent high‐error rebounds. Even after the optimization enters a relatively low‐error regime, large‐error samples continue to appear in later iterations. This prevents the surrogate model from stably concentrating on the truly low‐error and manufacturable region of the parameter space, ultimately constraining convergence. In contrast, BO‐CNN (Figure 4b) stabilizes on a low‐error plateau after approximately 25 evaluations and reaches the optimum at the 36th evaluation. This demonstrates that CNN‐based morphology screening markedly improves both optimization efficiency and robustness, achieving convergence nearly four times faster than BO without CNN filtering. This gain arises from two synergistic mechanisms. First, surrogate‐guided adaptive sampling initially explores the parameter space broadly to capture the overall response landscape and subsequently concentrates on low‐error regions that are physically plausible and sensitive to the target metric. Second, CNN‐based morphology feedback penalizes defective or non‐converged outcomes, thereby discouraging subsequent BO sampling in morphology‐invalid regions. As a result, the search can more rapidly concentrate on parameter regions that are both morphologically valid and consistent with the experimental observations. A closer inspection of the trajectory reveals an overall downward trend with occasional fluctuations, but without the severe oscillations observed in the CNN‐free setting. This indicates that, as informative evaluations accumulate, the surrogate becomes progressively more effective at targeting promising regions of the parameter space. Occasional high‐error evaluations reflect continued exploration of the stability of the low‐error basin and help mitigate premature convergence to a local optimum. Under identical BO settings, we further compared the optimization performance of different surrogate models. As shown in Figure S3, RF achieved calibration accuracy comparable to that of the automatic relevance determination (ARD) Matérn‐5/2 Gaussian process (GP) while yielding fewer high‐error and defect‐related evaluations than both GP variants, indicating higher overall search efficiency. Detailed surrogate‐model comparisons are provided in the Supporting Information.

**FIGURE 4:**
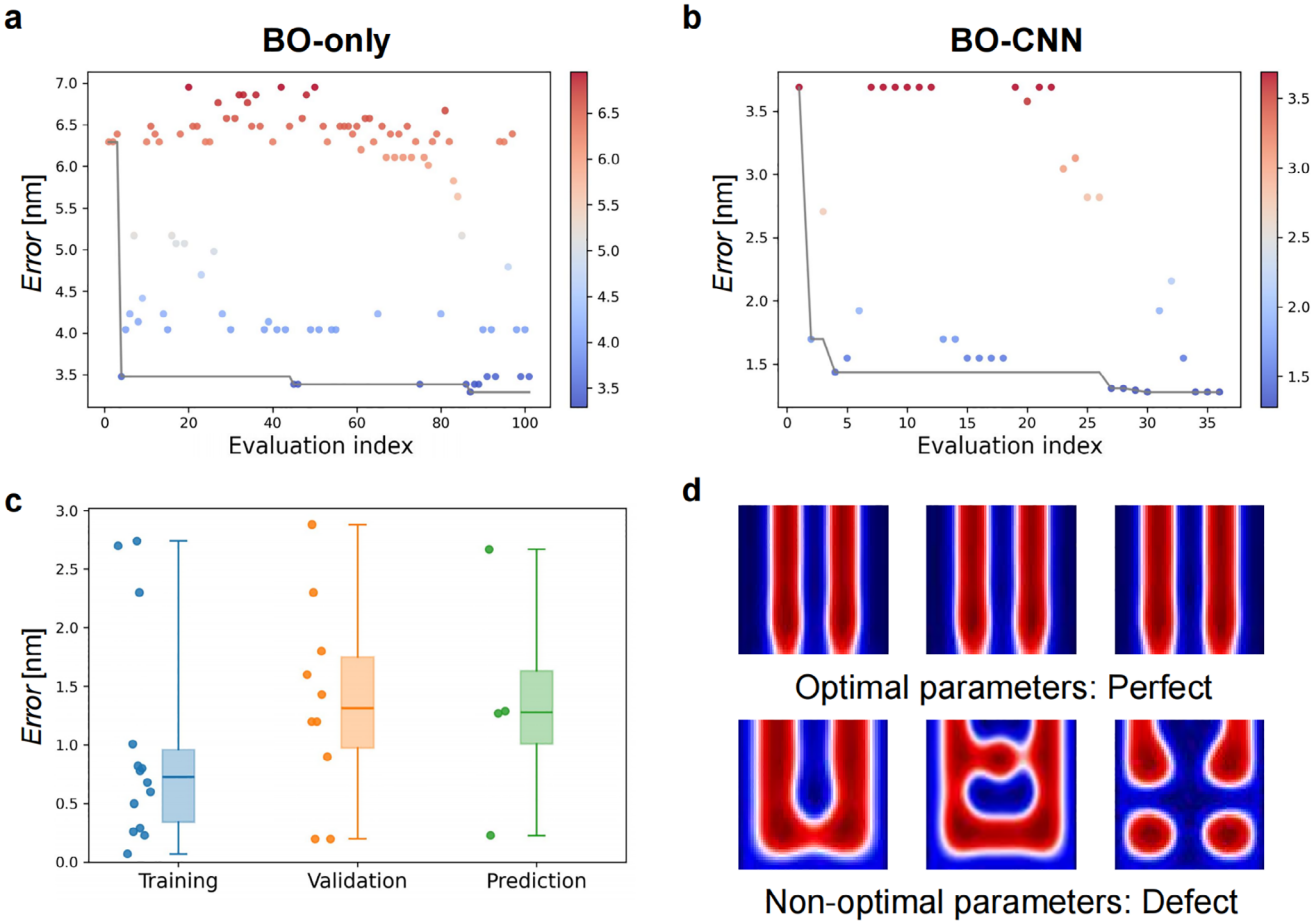
AI‐driven optimization and performance evaluation. (a) Evolution of the objective error during BO‐only optimization. (b) Evolution of the objective error during BO‐CNN‐assisted optimization. Scatter points represent the error obtained from each evaluated candidate, while the gray step line denotes the best‐so‐far error. Initial samples are excluded. (c) Error distributions across the training, validation and prediction datasets. (d) Representative simulated morphologies under optimal and non‐optimal parameter sets.

In addition, physically informed parameter bounds and empirically seeded initial samples reduce uninformative searches and accelerate convergence. Figure S1 shows that all parameters remain within the prescribed bounds throughout the optimization, exhibiting mild fluctuations without signs of divergence. After approximately 25 evaluations, sampling concentrates predominantly in the low‐error region. The degree of concentration differs across parameters: ϕ_*s*_ and Λ_*b*_(***r***) exhibit narrower ranges, indicating higher objective sensitivity, whereas ϕ_*b*_ and Λ_*s*_(***r***) remain more broadly distributed and are comparatively less influential. Importantly, the low‐error solutions do not correspond to a single point in parameter space but instead occupy a stable interval.

To further elucidate the error evolution and parameter‐search behavior, we analyzed the acquisition‐function values at the recommended sampling points as a function of evaluation index (Figure S2). The acquisition function reflects the expected potential for improvement in the parameter space. Higher values indicate regions that warrant further exploration, whereas lower values suggest that the corresponding region has been largely explored. During the early optimization stage, approximately evaluations 5–15, the acquisition‐function values fluctuate substantially, indicating a global exploration phase with relatively high model uncertainty and pronounced variations in both errors and parameters. Around the 20th evaluation, the acquisition‐function values increase again, suggesting that the model identifies additional promising regions for exploration. After approximately the 25th evaluation, the acquisition‐function values decrease and stabilize, indicating that the surrogate model has captured the objective landscape more accurately. The search consequently transitions from global exploration to localized refinement near the optimal region, with the error stabilizing on a low‐error plateau and the parameters gradually converging. Overall, the evolution of acquisition‐function values is consistent with the observed error convergence and parameter stabilization, indicating that the BO process shifts from exploration to exploitation and ultimately converges.

The optimal parameter set was identified as ϕ_*s*_ =  0.011, ϕ_*b*_ =  0.204, Λ_*s*_(***r***)  =  2.05 and Λ_*b*_(***r***)  =  3.29 by the automated workflow. Table S3 summarizes the template geometries, experimental CCD values and corresponding simulated CCD results, together with the associated errors for the training, validation and prediction sets. The simulations show excellent agreement with the experimental results and maintain high predictive accuracy on the independent prediction set. Figure 4c shows that the absolute CCD error across all 28 cases remains below 3 nm, with an average error of approximately 1.5 nm for the four prediction cases. Beyond minimizing numerical error, the optimized parameters also reveal a slightly stronger PS affinity at the bottom than at the sidewalls, consistent with experimental observations, thereby confirming the physical plausibility and interpretability of the solution. Representative cross‐sectional morphologies obtained using two different parameter sets are shown in Figure 4d. With the optimal parameter set, well‐defined and continuous PMMA cylinders were obtained, in agreement with the experimental observations. In contrast, non‐optimal parameter sets led to defective morphologies, such as cylinder breakage and bridging. These results demonstrate that the combined BO‐CNN screening strategy can efficiently and reliably identify physically valid parameter sets while excluding defective structures, enabling quantitative and morphological alignment between simulations and experiments.

To assess the predictive fidelity of CCD trends, two one‐dimensional (1D) geometry sweeps were conducted by varying CDY at fixed CDX = 68 nm and by varying CDX at fixed CDY = 127 nm, yielding the CCD response curves shown in Figure S14. The results reveal anisotropic CCD sensitivity. To reach the same CCD range of 50–56 nm, the required change in CDX is approximately three times larger than that in CDY, indicating higher sensitivity to CDY. Consistently, CCD increases with CDY with only minor fluctuations, whereas its dependence on CDX is comparatively weak. These trends agree with the experimental observations, supporting the reliability of the model in capturing CCD variations within the process window.

To further assess the predictive capability of the calibrated model, 10 racetrack‐template samples with different wafer locations and template dimensions were selected from an independent wafer for forward prediction. As shown in Table S4, with the calibrated parameters kept fixed, the model achieved a mean absolute CCD error of 0.55 nm, with all deviations below 1.5 nm and a maximum error of 1.31 nm. Further details are provided in the Supporting Information.

### 3.2 Inverse Design of DSA Templates Under CCD Constraint

Building on the forward‐predictive capability, we establish a CCD‐constrained inverse‐design framework based on the calibrated OK model. Rather than targeting a single optimal template, as in conventional inverse‐design approaches [24, 25, 26, 27], we systematically sweep the template geometry within the experimentally accessible process window to identify sampled manufacturable solutions that satisfy the same CCD target, thereby defining a feasible solution region. This region provides critical flexibility for layout decomposition algorithms. When routing conflicts arise or the process window is narrow, the decomposition tool can dynamically select different (CDX, CDY) combinations within this region, rather than being constrained to a single, potentially unmanufacturable solution.

To validate the manufacturability and experimental consistency of the inverse‐design outcomes, we imposed a target CCD of 52 ± 1 nm and performed a geometry sweep over track‐shaped templates spanning CDX = 50–80 nm and CDY = 110–130 nm. Figure 5a highlights the feasible region satisfying the CCD constraint. A stable CCD of 52 ± 1 nm was obtained for CDX = 52–80 nm and CDY = 120–128 nm, defining an effective solution domain for inverse template design. The existence of this domain indicates that multiple geometric combinations can generate CCD‐equivalent structures within the process tolerance. From this solution set, four representative templates were selected for experimental validation, with points 1 and 4 located at the domain boundaries and points 2 and 3 located within the interior. The simulated template geometries were translated into layout design parameters to generate the experimental template patterns. The corresponding layout parameters are summarized in Table 1, where CDX denotes the short‐axis dimension of the template, pitch represents the template spacing and R is the aspect ratio defined as CDY/CDX. The templates were fabricated according to the layout design, followed by DSA experiments and comparison with simulation predictions. Figure 5b shows that all four templates produced stable double‐hole morphologies in both simulations and experiments. The measured CCD values (Table 1) cluster around 52 nm, with simulation‐experiment deviations below 2 nm. To further validate the model, we shifted the target CCD to 54 ± 1 nm and tested an additional stable template solution with the smallest simulation error (Figure S15). A stable double‐hole morphology was again obtained, and the measured CCD remains centered near 54 nm, with deviations below 1.5 nm (Table 1).

**FIGURE 5:**
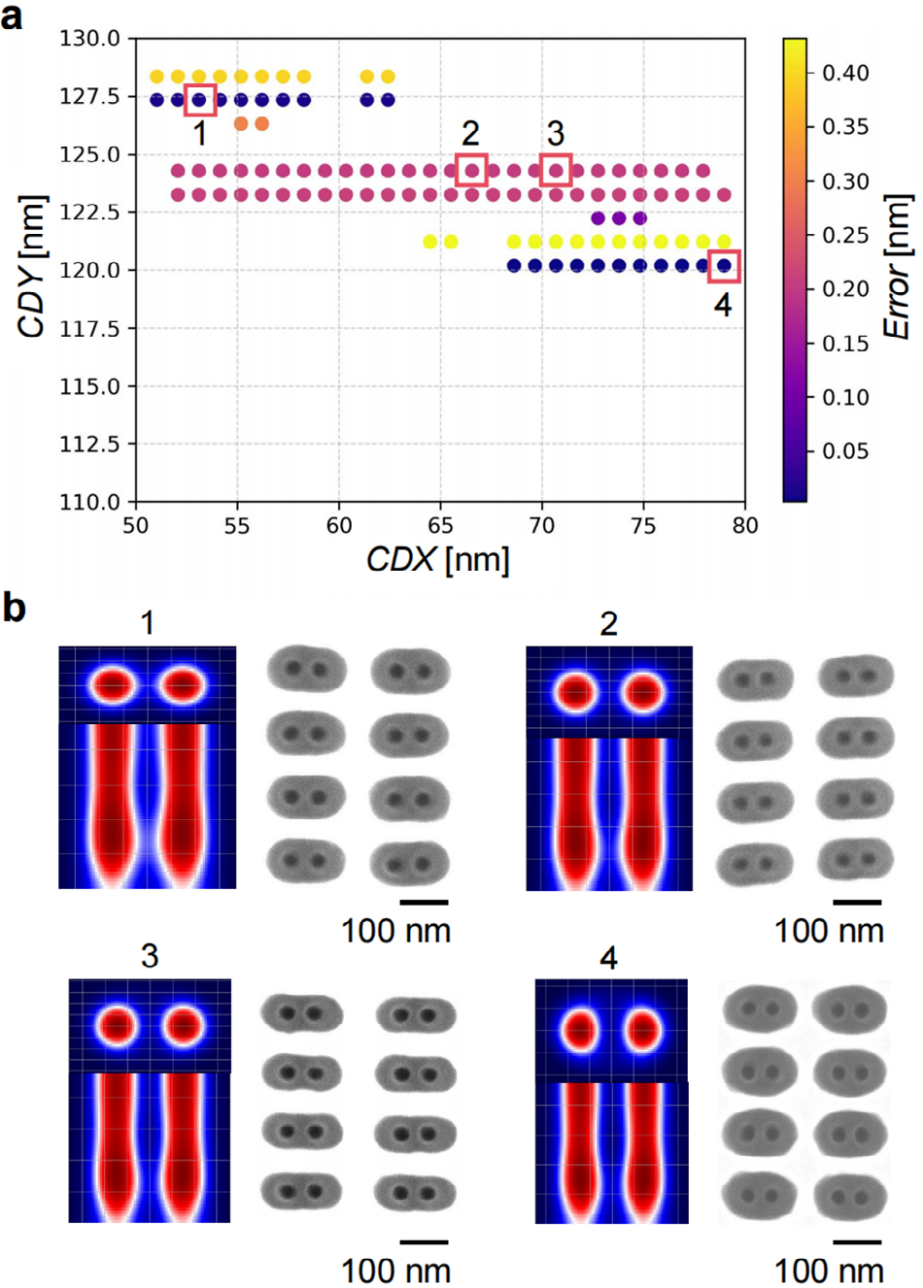
Inverse‐design window of DSA templates under CCD constraints and experimental validation. (a) Template design window satisfying CCD = 52 ± 1 nm. (b) Simulated and experimental morphologies for four representative template geometries. Scale bars are provided for reference.

**TABLE 1:** Experimental validation of inverse‐designed templates translated from simulation to layout implementation.

Case	Simulation			Layout design^a^			Experiment	Absolute error^b^ [nm]
	CDX [nm]	CDY [nm]	CCD [nm]	CDX [nm]	Pitch [nm]	R	CCD.[nm]	
**CCD = 52 nm**								
1	53	127	51.82	59	112	2.7	53.70	1.88
2	66	124	52.24	69	116	2.1	50.60	1.64
3	71	124	52.11	70	114	2.0	51.30	0.81
4	79	120	51.73	80	124	1.6	51.40	0.33
**CCD = 54 nm**								
1	62	129	54.45	64	110	2.4	55.50	1.05

^a^ The layout design parameters correspond to the template dimensions used for layout implementation, where CDX denotes the short‐axis dimension of the track‐shaped template, pitch represents the template spacing and *R* is the aspect ratio defined as CDY/CDX;

^b^ Absolute error denotes the deviation between simulation and experiment and is calculated as ∣Δ*CCD*∣  =  ∣*CCD*_exp_ − *CCD*_sim_∣.

To assess the robustness of the inverse‐design process windows at the two CCD targets with respect to variations in the calibrated parameters, three additional parameter sets were selected from the near‐converged low‐error plateau of the BO trajectory and used to recalculate the design windows. As shown in Figure S16, the four resulting feasible regions exhibit substantial overlap, and the intersection of all four feasible regions retains every experimentally validated template point. This indicates that the predicted process windows remain stable despite the non‐uniqueness of the calibrated parameters.

Across the two validation sets, the proposed CCD‐constrained inverse‐design framework demonstrates reliable predictive accuracy and experimental manufacturability. The calibrated OK model therefore supports target‐CCD‐driven inverse design for identifying multiple viable template solutions, providing increased flexibility and tolerance for template selection in layout decomposition. Instead of supplying EDA tools with a single deterministic design rule, this approach encodes DSA process constraints as a solution‐space map, thereby providing a basis for DTCO.

### 3.3 Extension of the Model to Peanut‐Shaped Templates

To further extend the applicability of the optimized model and broaden the template‐design space, we applied the model to peanut‐shaped templates. Without changing the process flow or re‐optimizing the model parameters, we evaluated its predictive capability against experimental data.

To accurately describe the geometric features of peanut‐shaped templates and establish a computable design space for template optimization, we constructed a parameterized representation (Figure 6). The template consists of two semicircular lobes connected by a central constricted region and is defined by five parameters (CDX, CDY, CW, Angle1 and Angle2). The semicircular lobes determine the template short axis (CDX) and long axis (CDY), whereas the central region is described by a Gaussian function:

(3) $$y\;\left(x\right)=A\exp\left(-\frac{{\left(x-x_{0}\right)}^{2}}{2\sigma^{2}}\right)$$

which generates a smooth constricted boundary. The parameters *A* and σ control the depth and width of the constriction, respectively, which in turn determine the neck width (CW) and the transition angles on the two sides (Angle1 and Angle2).

**FIGURE 6:**
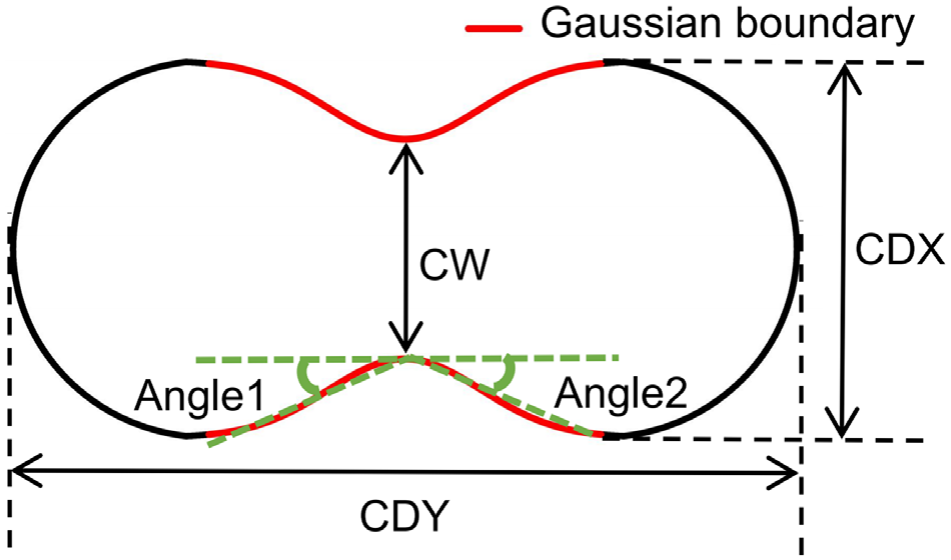
Parameterized representation of the peanut‐shaped template geometry.

Based on the parameterized template geometry, six experimental structures were selected for validation. Five geometric parameters of the experimental templates were extracted from SEM images, and the corresponding CCD values of the DSA structures were measured. The results are summarized in Table 2. Simulation templates were then constructed using the extracted geometric parameters to ensure close agreement with the experimental templates. For Case 1, the two sides of the experimental template exhibited slightly different measured angles; therefore, the average value was used as the connecting‐region angle in the simulation template. The experimental template morphology, parameterized template geometry and the resulting experimental and simulated morphologies are shown in Figure 7. Morphological comparison shows that the simulations reproduce the double‐hole features observed experimentally. Cross‐sectional views (Figure S17) further confirm the formation of vertically continuous cylindrical domains. CCD comparison (Table 2) shows good agreement between simulations and experiments, with a maximum deviation below 2.5 nm and an average error below 1.2 nm.

**TABLE 2:** Geometric parameters of parameterized peanut‐shaped templates and comparison between experimental and simulated CCD values.

Case	Experiment						Simulation	Absolute error^a^ [nm]
	CDX [nm]	CDY [nm]	CW [nm]	Angle1 [°]	Angle2 [°]	CCD [nm]	CCD.[nm]	
1	58.2	125.1	45.2	14.6	10.8	55.8	57.2	1.4
2	54.4	132.2	38.2	16.8	17.6	65.1	64.2	0.9
3	56.3	143.1	36.5	16.4	16.3	75.7	75.1	0.6
4	56.6	156.3	34.4	13.6	13.6	84.1	86.5	2.4
5	63.0	122.8	48.5	10.3	8.4	48.6	49.1	0.5
6	62.9	134.7	47.2	10.8	9.0	55.3	54.3	1.0

^a^ Absolute error is calculated as ∣Δ*CCD*∣  =  ∣*CCD*_exp_ − *CCD*_sim_∣.

**FIGURE 7:**
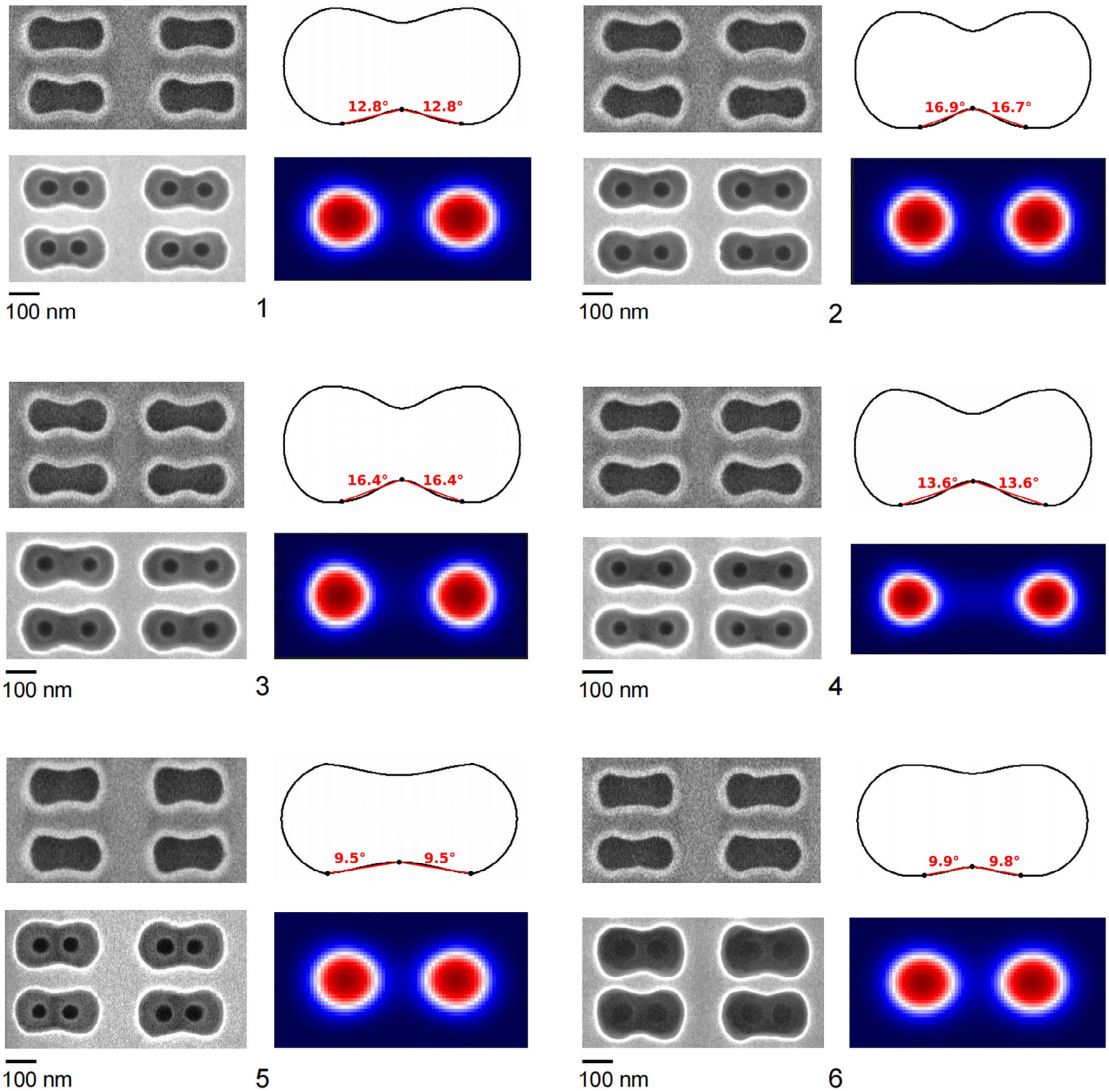
Experimental and simulated peanut‐shaped templates and the corresponding DSA morphologies. Scale bars are provided for reference.

Taken together, these results delineate the transferability boundary of the calibrated framework. Within the same material system and under comparable process conditions, the calibrated model parameters can be directly transferred to new template geometries, as evidenced by both the independent racetrack validation and the peanut‐shaped templates, while maintaining accurate prediction of DSA morphologies and key quantitative metrics such as CCD. Should the target morphology or task change substantially, the CNN classifier must be updated using task‐specific morphology data to accommodate new target structures and defect categories, although the underlying physics model and the Bayesian calibration workflow remain intact. If the BCP material system or key process conditions shift significantly, parameter recalibration against additional experimental data becomes necessary. In all of these scenarios, however, the overall framework requires no reconfiguration, thereby continuing to support rapid evaluation and co‐design across different template morphologies.

## 4 Conclusion

This work establishes an AI‐driven closed‐loop framework linking adaptive experimental calibration, forward prediction and template inverse design for DSA. By combining BO with CNN‐based morphology classification, defective or non‐converged outcomes are converted into penalty feedback for surrogate updates, preventing invalid CCD metrics from biasing parameter optimization. Compared with BO alone, the integrated strategy improves convergence efficiency and calibration accuracy while reducing dependence on manual parameter tuning. The calibrated 3D OK model achieves stable CCD predictions across different racetrack‐template dimensions, with an average simulation‐experiment deviation below 1.5 nm.

The results also clarify the complementary roles of CNNs and multimodal large language models in morphology screening. The trained CNN provides higher classification accuracy and inference efficiency, whereas LLMs can adapt to new image modalities, viewing perspectives, and defect criteria without additional task‐specific fine‐tuning by using text‐defined rules and a small number of reference examples. LLMs therefore provide a flexible supplementary tool when screening tasks or evaluation standards change.

Based on the calibrated model, forward simulation enables virtual assessment of template manufacturability before experiments, helping identify defect‐prone designs and reduce experimental trial and error. CCD‐constrained inverse design further generates multiple feasible template solutions within the process window, providing functionally equivalent candidates for DSA‐aware layout decomposition. The calibrated parameters can also be transferred to peanut‐shaped templates without additional optimization, achieving accurate morphology reproduction and maintaining an average prediction error below 1.2 nm. This cross‐geometry performance demonstrates the reusability of the calibrated model and expands the available design space for multi‐morphology applications.

Overall, the framework provides a reusable, simulation‐based pathway for incorporating DSA process constraints into layout design and DTCO. Future work will explore the joint integration of CNNs and multimodal LLMs into the BO loop, including LLM‐assisted CNN dataset expansion and more flexible morphology feedback, while incorporating thermal perturbations, extending inverse design to complex curvilinear templates through non‐parametric shape optimization, and integrating the framework with EDA tools for bidirectional layout‐process optimization.

## Author Contributions

**Caiwei Shang**: conceptualization, methodology, software, data curation, investigation, validation, formal analysis, visualization, writing – original draft, writing – review and editing. **Yanting Xu**: data curation, investigation, visualization, validation. **Yue Zhang**: investigation, data curation, visualization, validation. **Yan Zhang**: supervision, project administration, resources. **Jianfeng Li**: writing – review and editing, project administration, supervision. **Ming Li**: data curation. **Longlin Lei**: validation. **Wenda Bao**: data curation. **Weixuan Zeng**: supervision, project administration, resources, writing – review and editing, funding acquisition. **Shisheng Xiong**: funding acquisition, writing – review and editing, supervision, project administration, resources.

## Funding

This work was supported by the National Key Research and Development Program of China (No. 2024YFA1209000).

## Conflicts of Interest

The authors declare no conflicts of interest.

## Supporting information




**Supporting File**: advs78401‐sup‐0001‐SuppMat.pdf.

## Data Availability

The data and research materials that support the findings of this study are openly available in the associated GitHub repository at https://github.com/caiwei1126/AI‐Driven‐DSA‐DTCO. Additional methodological and computational details are provided in the Supporting Information.

## References

[1] V. Calado , S. G. J. Mathijssen , E. van Setten , et al., “Focus Sensing Using Placement and CD Variation for High NA EUV Lithography,” in Optical and EUV Nanolithography XXXVII, Proceedings of SPIE (SPIE, 2024), 1295307, 10.1117/12.3010835.

[2] H. J. Levinson , “Challenges and limits to patterning using extreme ultraviolet lithography,” Journal of Micro/Nanopatterning, Materials, and Metrology 24, no. 1 (2025): 011005, 10.1117/1.JMM.24.1.011005.

[3] N. Pellens , J.‐H. Franke , I. Lee , et al., “Imaging Roadmap for High‐NA EUV: Paving the Way to Single‐Patterning of Metal Logic for Future Nodes,” in Optical and EUV Nanolithography XXXIX, Proceedings of SPIE (SPIE, 2026), 139791J, 10.1117/12.3091514.

[4] Z. Levinson , Y. Zhang , L. Wu , et al., “Full Field Stitching‐Aware High‐NA EUV OPC/RET Flow,” in DTCO and Computational Patterning IV, Proceedings of SPIE (SPIE, 2025), 134250H, 10.1117/12.3052709.

[5] K. Lee , K. H. Kim , E. Vargo , G. S. W. Craig , R. Ruiz , and P. F. Nealey , “Directed Self‐Assembly of Block Copolymers for High‐Precision Patterning in the Era of Extreme Ultraviolet Lithography,” MRS Communications 15, no. 6 (2025): 1269–1288, 10.1557/s43579-025-00841-7.

[6] Y.‐T. Lin and I. H.‐R. Jiang , “Novel Guiding Template and Mask Assignment for DSA‐MP Hybrid Lithography Using Multiple BCP Materials,” IEEE Transactions on Computer‐Aided Design of Integrated Circuits and Systems 40, no. 9 (2021): 1909–1919, 10.1109/TCAD.2020.3033745.

[7] M. Muramatsu , T. Nakano , T. Tomita , K. Yamamoto , K. Matsuzaki , and T. Kitano , “Simulation Analysis of Directed Self‐Assembly for Hole Multiplication in Guide Pattern,” in Alternative Lithographic Technologies VI, Proceedings of SPIE (SPIE, 2014), 904921, 10.1117/12.2045865.

[8] M. Muramatsu , T. Nishi , G. You , et al., “Pattern Fidelity Improvement of Chemo‐Epitaxy DSA Process for High‐Volume Manufacturing,” in Alternative Lithographic Technologies VIII, Proceedings of SPIE (SPIE, 2016), 97770F, 10.1117/12.2218595.

[9] J. Doise , J. H. Koh , J. Y. Kim , et al., “Strategies for Increasing the Rate of Defect Annihilation in the Directed Self‐Assembly of High‐χ Block Copolymers,” ACS Applied Materials & Interfaces 11, no. 51 (2019): 48419–48427, 10.1021/acsami.9b17858. 31752485

[10] T. Iwama , N. Laachi , K. T. Delaney , et al., “The Hole Shrink Problem: Directed Self‐Assembly Using Self‐Consistent Field Theory,” Journal of Photopolymer Science and Technology 26, no. 1 (2013): 15–20, 10.2494/photopolymer.26.15.

[11] N. Laachi , T. Iwama , K. T. Delaney , D. Shykind , C. J. Weinheimer , and G. H. Fredrickson , “Directed Self‐Assembly of Linear Arrays of Block Copolymer Cylinders,” Journal of Polymer Science Part B: Polymer Physics 53, no. 5 (2015): 317–326, 10.1002/polb.23630.

[12] H. Wang , S. Li , J. Zeng , and T. Zhang , “Accelerating Polymer Self‐Consistent Field Simulation and Inverse DSA‐Lithography With Deep Neural Networks,” The Journal of Chemical Physics 162, no. 10 (2025): 104105, 10.1063/5.0255288. 40062757

[13] F. A. Detcheverry , G. Liu , P. F. Nealey , and J. J. de Pablo , “Interpolation in the Directed Assembly of Block Copolymers on Nanopatterned Substrates: Simulation and Experiments,” Macromolecules 43, no. 7 (2010): 3446–3454, 10.1021/ma902332h.

[14] T. Nakano , M. Matsukuma , K. Matsuzaki , M. Muramatsu , T. Tomita , and T. Kitano , “Dissipative Particle Dynamics Study on Directed Self‐Assembly in Holes,” in Alternative Lithographic Technologies V, Proceedings of SPIE (SPIE, 2013), 86801J, 10.1117/12.2011069.

[15] H. Sato , H. Yonemitsu , Y. Seino , et al., “Dissipative Particle Dynamics Simulations to Optimize Contact Hole Shrink Process Using Graphoepitaxial Directed Self‐Assembly,” in Alternative Lithographic Technologies V, Proceedings of SPIE (SPIE, 2013), 86801K, 10.1117/12.2009622.

[16] K. Yoshimoto and T. Taniguchi , “Large‐Scale Dynamics of Directed Self‐Assembly Defects on Chemically Pre‐Patterned Surface,” in Alternative Lithographic Technologies V, Proceedings of SPIE (SPIE, 2013), 86801I, 10.1117/12.2011826.

[17] K. Yoshimoto , K. Fukawatase , M. Ohshima , et al., “Optimization of Directed Self‐Assembly Hole Shrink Process With Simplified Model,” Journal of Micro/Nanolithography, MEMS, and MOEMS 13, no. 3 (2014): 031305, 10.1117/1.JMM.13.3.031305.

[18] C. Shang , J. Luo , W. Zeng , Y. Zhang , and S. Xiong , “Fast Simulation of Directed Self‐Assembly for Contact Hole Multiplication With an Optimized Compact Model,” in Ninth International Workshop on Advanced Patterning Solutions (IWAPS 2025), Proceedings of SPIE (SPIE, 2025), 1399112, 10.1117/12.3093234.

[19] L. Cao , K. Wu , J. T. Oden , P. Chen , and O. Ghattas , “Bayesian Model Calibration for Diblock Copolymer Thin Film Self‐Assembly Using Power Spectrum of Microscopy Data and Machine Learning Surrogate,” Computer Methods in Applied Mechanics and Engineering 417 (2023): 116349, 10.1016/j.cma.2023.116349.

[20] K. Lai , M. Ozlem , J. W. Pitera , et al., “Computational Lithography Platform for 193i‐Guided Directed Self‐Assembly,” in Optical Microlithography XXVII, Proceedings of SPIE (2014), 90521A, 10.1117/12.2046920.

[21] C.‐C. Liu , J. Pitera , N. Lafferty , et al., “Progress Towards the Integration of Optical Proximity Correction and Directed Self‐Assembly of Block Copolymers With Graphoepitaxy,” in Alternative Lithographic Technologies IV, Proceedings of SPIE (SPIE, 2012), 83230X, 10.1117/12.916525.

[22] T. Fühner , U. Welling , M. Müller , and A. Erdmann , “Rigorous Simulation and Optimization of the Lithography/Directed Self‐Assembly Co‐Process,” in Optical Microlithography XXVII, Proceedings of SPIE (SPIE, 2014), 90521C, 10.1117/12.2047381.

[23] Y. Ma , Y. Wang , J. Word , et al., “Directed Self Assembly (Dsa) Compliant Flow With Immersion Lithography: From Material to Design and Patterning,” in Alternative Lithographic Technologies VIII, Proceedings of SPIE (SPIE, 2016), 97770N, 10.1117/12.2219505.

[24] A. Latypov , “Computational Solution of Inverse Directed Self‐Assembly Problem,” in Alternative Lithographic Technologies V, Proceedings of SPIE (SPIE, 2013), 86800Z, 10.1117/12.2011575.

[25] G. Y. Ouaknin , N. Laachi , K. T. Delaney , G. H. Fredrickson , and F. Gibou , “Level‐Set Strategy for Inverse DSA‐Lithography,” Journal of Computational Physics 375 (2018): 1159–1178, 10.1016/j.jcp.2018.09.021.

[26] D. Bochkov and F. Gibou , “A Non‐Parametric Gradient‐Based Shape Optimization Approach for Solving Inverse Problems in Directed Self‐Assembly of Block Copolymers,” Communications on Applied Mathematics and Computation 6, no. 2 (2024): 1472–1489, 10.1007/s42967-024-00394-x.

[27] A. F. Hannon , Y. Ding , W. Bai , C. A. Ross , and A. Alexander‐Katz , “Optimizing Topographical Templates for Directed Self‐Assembly of Block Copolymers via Inverse Design Simulations,” Nano Letters 14, no. 1 (2014): 318–325, 10.1021/nl404067s. 24328687

[28] A. F. Hannon , K. W. Gotrik , C. A. Ross , and A. Alexander‐Katz , “Inverse Design of Topographical Templates for Directed Self‐Assembly of Block Copolymers,” ACS Macro Letters 2, no. 3 (2013): 251–255, 10.1021/mz400038b. 35581891

[29] S. Shim and Y. Shin , “Mask Optimization for Directed Self‐Assembly Lithography: Inverse DSA and Inverse Lithography,” in 2016 21st Asia and South Pacific Design Automation Conference (ASP‐DAC) (IEEE, 2016), 83–88, 10.1109/ASPDAC.2016.7427993.

[30] C. Ma , Y. Wang , D. Zhang , et al., “Closed‐Loop Discovery of Energy Materials Empowered by Artificial Intelligence Models,” Digital Discovery 5, no. 8 (2026): 3151–3157, 10.1039/D6DD00218H.

[31] Z. Liu , Y.‐X. Liu , Y. Yang , and J. Li , “Template Design for Complex Block Copolymer Patterns Using a Machine Learning Method,” ACS Applied Materials & Interfaces 15, no. 25 (2023): 31049–31056, 10.1021/acsami.3c05018. 37335810

[32] G. S. Doerk , A. Stein , S. Bae , M. M. Noack , M. Fukuto , and K. G. Yager , “Autonomous Discovery of Emergent Morphologies in Directed Self‐Assembly of Block Copolymer Blends,” Science Advances 9, no. 2 (2023): add3687, 10.1126/sciadv.add3687. PMC9839324 36638174

[33] Q. Dong , X. Gong , K. Yuan , Y. Jiang , L. Zhang , and W. Li , “Inverse Design of Complex Block Copolymers for Exotic Self‐Assembled Structures Based on Bayesian Optimization,” ACS Macro Letters 12, no. 3 (2023): 401–407, 10.1021/acsmacrolett.3c00020.34. 36888723

[34] Q. Dong , Z. Xu , Q. Song , Y. Qiang , Y. Cao , and W. Li , “Automated Search Strategy for Novel Ordered Structures of Block Copolymers,” ACS Macro Letters 13, no. 8 (2024): 987–993, 10.1021/acsmacrolett.4c00384. 39042468

[35] S. Zhao , T. Cai , L. Zhang , W. Li , and J. Lin , “Autonomous Construction of Phase Diagrams of Block Copolymers by Theory‐Assisted Active Machine Learning,” ACS Macro Letters 10, no. 5 (2021): 598–602, 10.1021/acsmacrolett.1c00133. 35570770

[36] S. Cheon , H. Lee , C. O. Kim , and S. H. Lee , “Convolutional Neural Network for Wafer Surface Defect Classification and the Detection of Unknown Defect Class,” IEEE Transactions on Semiconductor Manufacturing 32, no. 2 (2019): 163–170, 10.1109/TSM.2019.2902657.

[37] F. L. de la Rosa , J. L. Gómez‐Sirvent , R. Morales , R. Sánchez‐Reolid , and A. Fernández‐Caballero , “A Deep Residual Neural Network for Semiconductor Defect Classification in Imbalanced Scanning Electron Microscope Datasets,” Applied Soft Computing 131 (2022): 109743, 10.1016/j.asoc.2022.109743.

[38] E. Dehaerne , B. Dey , V. Blanco , and J. Davis , “Scanning Electron Microscopy‐Based Automatic Defect Inspection for Semiconductor Manufacturing: A Systematic Review,” Journal of Micro/Nanopatterning, Materials, and Metrology 24, no. 02 (2025): 1–28, 10.1117/1.JMM.24.2.020901.

[39] H. Liu , C. Li , Q. Wu , and Y. J. Lee , “Visual Instruction Tuning,” Advances in Neural Information Processing Systems 36 (2023): 34892–34916, 10.52202/075280-1516. PMC11867732 40017809

[40] J.‐B. Alayrac , J. Donahue , P. Luc , et al., “Flamingo: A Visual Language Model for Few‐Shot Learning,” Advances in Neural Information Processing Systems 35 (2022), 23716–23736, 10.52202/068431-1723.

[41] P. Verma , M.‐H. Van , and X. Wu , “Vision Language Models for Scientific Image Analysis: An Evaluation Highlighting Opportunities and Challenges,” npj Computational Materials 12, no. 1 (2026): 218, 10.1038/s41524-026-02069-y. PMC13287007 42344950

[42] Y. Badr , J. A. Torres , Y. Ma , J. Mitra , and P. Gupta , “Incorporating DSA in Multipatterning Semiconductor Manufacturing Technologies,” in Design‐Process‐Technology Co‐Optimization for Manufacturability IX, Proceedings of SPIE, vol. 9427 (2015): 94270P, 10.1117/12.2084776.

[43] Y. Yang , W.‐S. Luk , H. Zhou , et al., “An Effective Layout Decomposition Method for DSA with Multiple Patterning in Contact‐Hole Generation,” ACM Transactions on Design Automation of Electronic Systems 23, no. 1 (2017): 1–27, 10.1145/3131847.

[44] T. Ohta and K. Kawasaki , “Equilibrium Morphology of Block Copolymer Melts,” Macromolecules 19, no. 10 (1986): 2621–2632, 10.1021/ma00164a028.

[45] R. Garnett , Bayesian Optimization (Cambridge University Press, 2023), 10.1017/9781108348973.

[46] H. Luo , Y. Cho , J. W. Demmel , I. Kozachenko , X. S. Li , and Y. Liu , “Non‐Smooth Bayesian Optimization in Tuning Scientific Applications,” The International Journal of High Performance Computing Applications 38, no. 6 (2024): 633–657, 10.1177/10943420241278981.

[47] Qwen Team , Qwen3.6‐Plus: Towards Real World Agents, (2026), https://qwen.ai/blog?id=qwen3.6, accessed June 9, 2026.

[48] GLM‐V Team, “GLM‐5V‐Turbo: Toward a Native Foundation Model for Multimodal Agents,” *arXiv* (2026): arXiv:2604.26752, 10.48550/arXiv.2604.26752.

[49] OpenAI , “Introducing GPT‐5.4,” (2026), https://openai.com/index/introducing‐gpt‐5‐4/, accessed June 9, 2026.

[50] Google AI for Developers , Gemini 3.1 Pro Preview, Gemini API Documentation (2026), https://ai.google.dev/gemini‐api/docs/models/gemini‐3.1‐pro‐preview, accessed June 9, 2026.

