# Supplementary material for: An AI‐Driven Framework for Directed Self‐Assembly Toward Design‐Technology Co‐Optimization
Source: Adv Sci (Weinh). 2026 Oct 22:e78401. Online ahead of print. doi: 10.1002/advs.78401 (PMC13691222; doi:10.1002/advs.78401)
Supplement: Supplementary file 1 [file ADVS-9999-e78401-s001.pdf]

Supporting Information

An AI-Driven Framework for Directed Self-Assembly toward Design-Technology Co-Optimization

Caiwei Shang, Yanting Xu, Yue Zhang, Yan Zhang, Jianfeng Li, Ming Li, Longlin Lei, Wenda Bao, Weixuan Zeng, Shisheng Xiong**

This Supporting Information file includes:

Ohta-Kawasaki (OK) model details.

Dynamic simulations.

Experimental procedure details.

Bayesian optimization (BO) details.

Convolutional neural network (CNN) details.

Multimodal large language models (LLMs) screening details.

External validation of the calibrated model using racetrack templates from an independent wafer.

Robustness analysis of the inverse-design process windows.

Key simulation, optimization, and CNN training settings.

Figure S1-S17.

Table S1-S8.

Table S1. Comparison of DSA design and inverse-design approaches with the present framework.

Representative studies	Main focus	Core approach	Experimental calibration	Design output
[20-23]	Forward co-optimization	Simulation-based optimization	No	Process/design conditions
[24-28]	Template recovery	Physics-based inverse optimization	No	Guiding template
[29]	Guide and mask optimization	Sequential inverse optimization	No	Guide and mask
[31]	Target-to-template mapping	Machine learning	No	Guiding template
**This work**	**Experiment-aligned prediction and inverse design**	**Physics model with AI-assisted calibration**	**Yes**	**Feasible design window**

1. Ohta-Kawasaki (OK) model details

The proposed framework is based on the Ohta-Kawasaki (OK) model.[S1] All length-related variables are scaled by the radius of gyration ($Rg = a\sqrt{\frac{N}{2}}$), where a is the Kuhn length of the diblock copolymer, and N is the degree of polymerization. The free energy of the bulk diblock copolymer, $\mathcal{F}$, is given as follows:

$$\mathcal{F}[\Psi(\boldsymbol{r})] = c_2\Psi(\boldsymbol{r})^2 + c_4\Psi(\boldsymbol{r})^4 + c_5[\nabla\Psi(\boldsymbol{r})]^2 + c_7\int \boldsymbol{dr'}G(\boldsymbol{r},\boldsymbol{r'})[\Psi(\boldsymbol{r}) - \bar{\Psi}][\Psi(\boldsymbol{r'}) - \bar{\Psi}] \quad \textbf{(S1)}$$

The order parameter, $\Psi(\boldsymbol{r})$, and the average order parameter, $\bar{\Psi}$, are defined as $\Psi(\boldsymbol{r}) = \phi_A(\boldsymbol{r}) - \phi_B(\boldsymbol{r}) = 2\phi_A(\boldsymbol{r}) - 1$ and $\bar{\Psi} = 2f - 1$, where $\phi_A(\boldsymbol{r})$ is the density fraction of the minor component, poly(methyl methacrylate) (PMMA), and f is the volume fraction of the PMMA. It is assumed that the system is incompressible.

In **Equation S1**, the terms on the right-hand side can be interpreted as follows: the first two contributions account for the mixing free energy, while the third term corresponds to the interfacial energy. The final term introduces long-range interactions between the order parameters through the Green's function $G(\boldsymbol{r},\boldsymbol{r'})$, which satisfies $-\nabla^2 G(\boldsymbol{r},\boldsymbol{r'}) = \delta(\boldsymbol{r} - \boldsymbol{r'})$ and $\boldsymbol{r'}$ denotes the position vector.

The coefficients c_2, c_4, c_5 and c_7 are model parameters. c_2 controls segregation strength. c_4 ensures that the free energy function has a finite value. c_5 characterizes the energetic penalty associated with spatial variations in the PMMA-polystyrene (PS) interface. c_7 represents the long-range interactions between them. The model parameters were set to $c_2 =$

-1.4, $c_4 = 0.25$ and $c_5 = c_7 = 0.5$ to form a stable cylindrical morphology in the bulk phase.[S2]

Beyond the bulk free energy $\mathcal{F}$, an additional surface energy term $\mathcal{F}_s$ is incorporated to capture the preferential interaction between the diblock copolymer and the chemically pre-patterned substrate:

$$\mathcal{F}_s[\Psi(\boldsymbol{r})] = \int \boldsymbol{dr}\{\Lambda_s(\boldsymbol{r})[\phi_A(\boldsymbol{r}) - \phi_s]^2 + \Lambda_b(\boldsymbol{r})[\phi_A(\boldsymbol{r}) - \phi_b]^2\} \quad \textbf{(S2)}$$

where $\phi_A(\boldsymbol{r})$ is the density fraction of the PMMA, while ϕ_s and ϕ_b denote the volume fractions of the selectively attracted block component of the diblock copolymers in the vicinity of the walls and the bottom, respectively. Compared with Ref. [S2], the present formulation extends the number of surface parameters from two to four to distinguish the bottom and sidewall interactions, leading to more accurate optimization and improved agreement with experimental results. Both ϕ_s and ϕ_b are smaller than 0.5, corresponding to PS-attractive surfaces, and were calibrated to match experimental observations. $\Lambda_s(\boldsymbol{r})$ and $\Lambda_b(\boldsymbol{r})$ are the position-dependent coupling parameters that determine the strength of the surface interaction. Larger values indicate stronger preferential interactions, while $\Lambda = 0$ corresponds to non-preferential regions.

2. Dynamic simulations

The cell dynamics method is employed to numerically calculate the time evolution of the order parameter in a three-dimensional (3D) simulation using the time-dependent Ginzburg-Landau (TDGL) equation.[S3,S4]

$$\frac{\partial \Psi(r,t)}{\partial t} = D\nabla^2\mu \quad \textbf{(S3)}$$

where t, D, and μ represent time, the diffusion constant, and the chemical potential, respectively.

The chemical potential, μ, is obtained as the functional derivative of the total free energy with respect to $\Psi(\boldsymbol{r},t)$, where the total free energy is the sum of the bulk and surface free energies given in **Equations** S1 and **S2**:

$$\mu = \frac{\delta[\mathcal{F} + \mathcal{F}_s]}{\delta\Psi(\boldsymbol{r},t)}$$

$$= -2.8\Psi(\boldsymbol{r},t) + \Psi(\boldsymbol{r},t)^3 - \nabla^2\Psi(\boldsymbol{r},t) + \int \boldsymbol{dr}' G(\boldsymbol{r},\boldsymbol{r}')[\Psi(\boldsymbol{r}') - \bar{\Psi}] + \Lambda_s(\boldsymbol{r})[\phi_A(\boldsymbol{r}) - \phi_s] + \Lambda_b(\boldsymbol{r})[\phi_A(\boldsymbol{r}) - \phi_b] \quad \textbf{(S4)}$$

By substituting **Equation S4** into **Equation S3**, the time-evolution equation of the order parameter is given by:

$$\Psi(\boldsymbol{r},t+\Delta t) \approx \Psi(\boldsymbol{r},t) + D\Delta t\{\nabla^2\mu_s(\boldsymbol{r},t) - [\Psi(\boldsymbol{r}',t) - \bar{\Psi}]\} \quad \textbf{(S5)}$$

with

$$\mu_s(\boldsymbol{r},t) = -2.8\Psi(\boldsymbol{r},t) + \Psi(\boldsymbol{r},t)^3 - \nabla^2\Psi(\boldsymbol{r},t) + \Lambda_s(\boldsymbol{r})[\phi_A(\boldsymbol{r}) - \phi_s] + \Lambda_b(\boldsymbol{r})[\phi_A(\boldsymbol{r}) - \phi_b] \quad \textbf{(S6)}$$

This work employs a 3D finite-difference grid to numerically solve the OK model, enabling simultaneous capture of both the in-plane morphology and the structural evolution along the vertical direction. The main numerical solution steps for **Equations S5 and S6** are as follows:

Step 1: 3D grid initialization.

The simulation box is divided into cubic grids, with a uniform grid spacing of $\Delta l = 0.2\ Rg$. Based on stability analysis of the numerical method, the maximum time step is chosen as $\Delta t = 2.2 \times 10^{-5}\ t_0$, where t_0 denotes the characteristic time unit.

Step 2: Calculate $\mu_s(\boldsymbol{r},t)$.

The order parameter $\Psi(\boldsymbol{r}, t = 0)$ is initialized in each grid cell by adding a small random perturbation, i.e., a uniformly distributed random value in the range from $\overline{\Psi} - 0.05$ to $\overline{\Psi} + 0.05$, to represent a weakly disordered initial state prior to simulated annealing. Then, the Laplacian of the initial order-parameter field, $\nabla^2\Psi(\boldsymbol{r},t)$, is computed using the standard 3D discrete central-difference scheme and substituted into Equation S6 to obtain $\mu_s(\boldsymbol{r},t)$. Neumann no-flux boundary conditions are imposed on the bottom, top, and sidewalls of the simulation box to ensure mass conservation.

Step 3: Update the order parameter $\Psi(\boldsymbol{r},t)$.

$\nabla^2\mu_s(\boldsymbol{r},t)$ is calculated using the standard 3D discrete central-difference scheme, and the order parameter $\Psi(\boldsymbol{r},t)$ is then updated in time according to Equation S5.

Step 4: Iterate the equations.

Steps 2 and 3 are repeated until the system reaches equilibrium, after which the final morphology and associated simulation outputs are collected.

3. Experimental procedure details

The directed self-assembly (DSA) hole-multiplication process used in our experiment was as follows:

(1) Guiding template preparation. A tetraethyl orthosilicate (TEOS) layer was first deposited on 300 mm silicon wafers to serve as a hard mask. A spin-on carbon (SOC) layer was subsequently coated using a Tokyo Electron CLEAN TRACK LITHIUS Pro Z^{TM} system, followed by silicon-containing antireflective coating (SiARC) deposition and photoresist coating and development on a Sokudo DUO track system. Pattern exposure was carried out using an ASML NXT:1950i immersion lithography scanner integrated with the track system. The patterned features were then transferred into the underlying SiARC and SOC layers via

dry etching using a Honghu Ivory system (Bangxin Semiconductor). Residual photoresist was removed using a QiJi Spinel 300A system. Template sidewall profiles were characterized by focused ion beam scanning electron microscopy (FIB-SEM) and transmission electron microscopy (TEM) (Helios 5 UX and Talos F200X S/TEM), while overall critical dimensions and pattern uniformity were analyzed using a Hitachi High-Tech critical-dimension scanning electron microscopy (CD-SEM, CV6300).

(2) Polymer brush grafting. A random copolymer brush (PS-*r*-PMMA, PS content ≈ 72%) was spin-coated using the CLEAN TRACK LITHIUS Pro ZTM system and thermally annealed at 200 °C for 3 min under a nitrogen atmosphere to achieve surface grafting. Excess ungrafted polymer was subsequently removed by rinsing with propylene glycol monomethyl ether acetate (PGMEA), resulting in a uniform grafted layer that provided preferential wetting for the PS domains.

(3) Block copolymer (BCP) thermal annealing. A cylinder-forming PS-b-PMMA ($L_0 \approx 42.5\ nm$, $f = 0.28$) thin film was spin-coated on the grafted template using the same track system and thermally annealed at 220 °C for 6 min in a nitrogen atmosphere. Microphase separation of the BCP was thereby induced, leading to the formation of ordered nanostructures guided by the underlying template.

(4) Selective PMMA removal. Selective removal of the PMMA domains was performed via dry etching using a Honghu Ivory system operated at 150 W. The resulting microphase-separated morphologies were characterized by FIB-SEM and TEM, while the DSA pattern fidelity was evaluated using CD-SEM imaging. The process window for double-hole multiplication was systematically analyzed through quantitative center-to-center distance (CCD) measurements using in-house-developed analysis software.

(5) Pattern transfer. The assembled DSA patterns were transferred into the TEOS hard mask layer through dry etching using the Honghu Ivory system (150 W). The transferred structures were characterized by CD-SEM, and pattern transfer quality was quantitatively evaluated using the in-house analysis platform.

4. Bayesian optimization (BO) details

In this work, a standard BO procedure is employed to efficiently search the four-dimensional design space of tunable template-BCP interaction parameters, $\mathbf{X} = (\phi_s, \phi_b, \Lambda_s(\boldsymbol{r}), \Lambda_b(\boldsymbol{r}))$.[S5] Accordingly, the objective function is defined as:

$$f(\boldsymbol{X}) = \begin{cases} |CCD_{sim}(\boldsymbol{X}) - CCD_{exp}|, & \text{valid sample,} \\ 1000, & \text{invalid sample.} \end{cases} \quad \textbf{(S7)}$$

Here, valid samples refer to converged simulations with morphologically valid outcomes, whereas defective or non-converged samples are treated as invalid. $CCD_{\mathrm{sim}}(\boldsymbol{X})$ denotes the simulated CCD obtained with parameter set $\boldsymbol{X}$, and CCD_{exp} is the experimentally measured CCD for the same template dimensions. Thus, $f(\boldsymbol{X})$ corresponds to the absolute simulation-experiment CCD error for valid samples, while invalid samples are assigned a fixed penalty value of 1000.

In practice, the optimization is initialized by sampling the four-dimensional parameter space using a Sobol low-discrepancy sequence, which ensures uniform global coverage and enhances exploratory capability in the early stages. To further accelerate convergence, a small number of empirically selected seed points are incorporated. The seed points are taken from the manually calibrated parameter sets reported in Ref. [S6], where systematic tuning of model parameters yielded good agreement between simulated and experimental morphologies. As the present study uses an experimental and simulation setup similar to that in Ref. [S6] while improving the optimization strategy, the parameters reported in Ref. [S6] serve as a physically interpretable starting reference that guides the search toward a reasonable region of the parameter space without compromising global exploration. The initial parameter bounds are defined based on physical constraints and prior knowledge, and they are progressively refined during optimization to exclude insensitive regions and unphysical boundary values. Specifically, the composition parameters ϕ_s and ϕ_b are restricted to the range [0, 0.5], consistent with preferential PS affinity at the template sidewalls and bottom surface observed experimentally. The surface-field strength parameters $\Lambda_s(\boldsymbol{r})$ and $\Lambda_b(\boldsymbol{r})$ are set to [0, 10], covering the transition from weak to strong template affinity. As the optimization progresses, the surrogate model gradually accumulates information about the objective function, and sampling contracts toward parameter subspaces that are both highly sensitive to the target metric and physically plausible, reducing uninformative evaluations and improving overall optimization efficiency.

The BO procedure requires evaluations of the objective function $f(\mathbf{X})$. At each iteration, BO calls the OK model to simulate the system under a proposed parameter set **X** and computes the corresponding $CCD_{\mathrm{sim}}(\mathbf{X})$. Not all queried parameter sets yield a valid objective value. Samples are treated as invalid if the simulation fails to converge or yields defective morphologies. As shown in **Figure S4**, non-convergence corresponds to $\phi_{\mathrm{max}} < 0.8$, indicating weak phase separation and an indistinguishable morphology. For invalid samples, the CCD error is not evaluated and a fixed penalty value of 1000 is assigned as the objective; for converged and morphologically valid samples, the simulation-experiment CCD error is

used instead. In both cases, the resulting parameter-objective pair is used to update the RF surrogate, allowing the surrogate to learn and progressively avoid invalid regions while guiding the search toward physically reasonable parameter regions. The optimization was allowed to proceed for up to 100 iterations and was manually terminated after reaching a stable low-error regime.

A Random Forest regressor (RF) is adopted as a nonparametric surrogate model, from which the predictive mean $\mu(\mathbf{X})$ and uncertainty $\sigma(\mathbf{X})$ are estimated using ensemble statistics.[S7] RF was selected as the surrogate model because its tree-based ensemble structure can flexibly represent nonlinear response landscapes and remains computationally efficient for sequential model-based optimization.[S8]

For sequential decision-making, the expected improvement (EI) acquisition function is used to select candidate points that maximize the expected gain relative to the current best objective value,[S9] thereby balancing exploitation of low-μ regions and exploration of high-σ regions. At each iteration, one candidate parameter set is selected using the EI acquisition function, evaluated by the OK simulation, and subsequently used to update the RF surrogate before the next candidate is proposed.

Figure S1 summarizes the search trajectories and convergence behavior of all parameters and reveals similar overall trends throughout the optimization. All candidate parameters remain within the prescribed empirical bounds (orange dashed lines), and no out-of-range excursions or divergence are observed. As iterations proceed, after approximately 25 evaluations, low-error solutions increasingly cluster within stable intervals of the parameter space, supporting the appropriateness of the chosen search domain and indicating a well-behaved model response over this range. However, the parameters exhibit nonuniform convergence, with differentiated concentration trends reflecting their relative influence on the objective. ϕ_s and $\Lambda_b(\boldsymbol{r})$ remain confined to narrow ranges across iterations, indicating that the objective is most sensitive to them. In contrast, ϕ_b and $\Lambda_s(\boldsymbol{r})$ retain a broader spread, suggesting a weaker impact on the final error and a larger feasible tuning margin. Notably, low-error solutions span a finite, continuous region in parameter space, rather than a single optimum, implying a tolerance window for the calibrated parameters, in agreement with experimental observations.

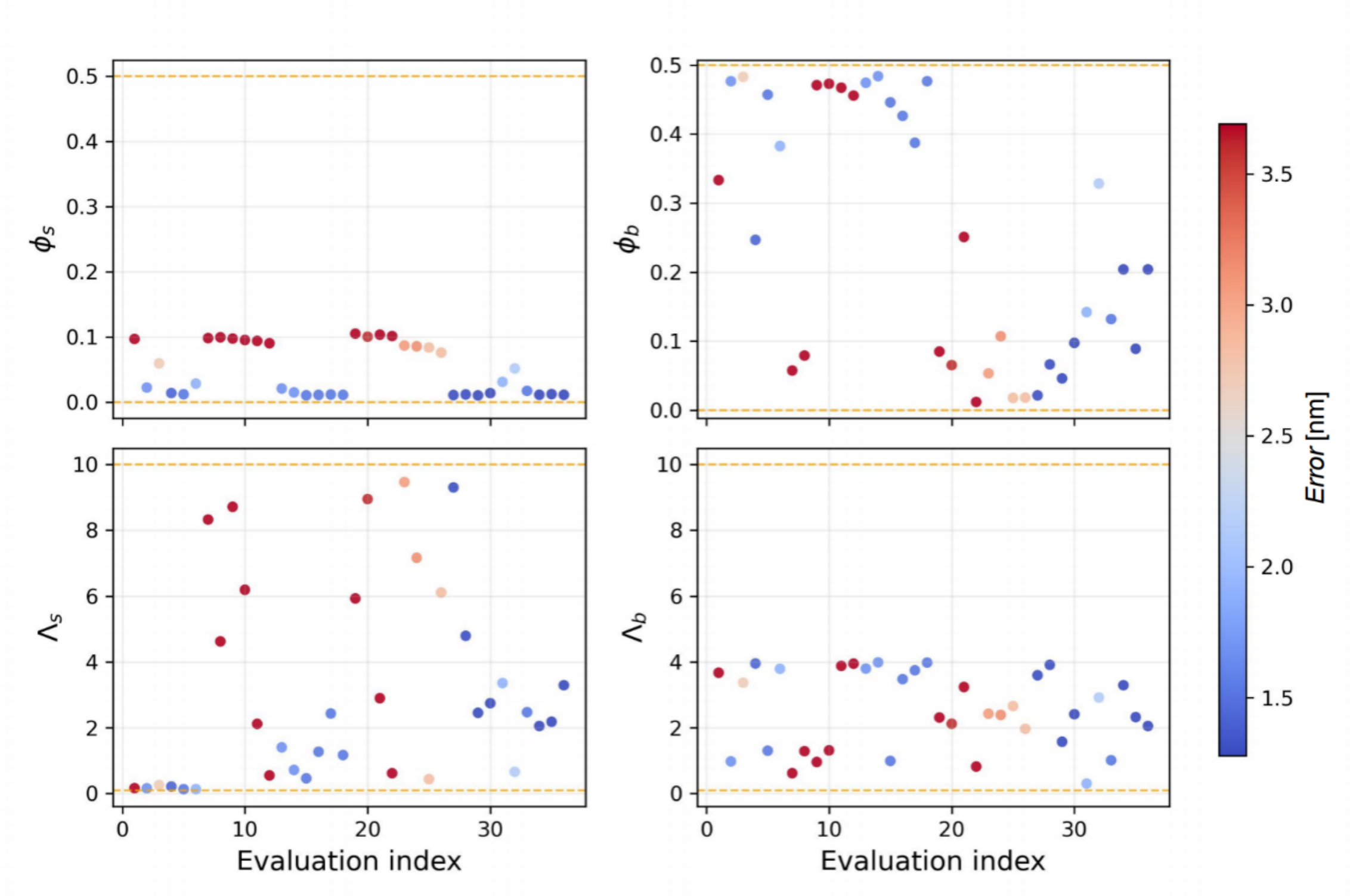

Figure S1. Evolution of model parameters during Bayesian optimization.

Figure S2 presents the evolution of the acquisition-function values evaluated at the sampled points during the BO process. The acquisition function reflects the EI estimated by the surrogate model for each recommended parameter set, quantifying the potential benefit of sampling a given location in the parameter space. Each data point corresponds to the acquisition-function value associated with the parameter set selected at a given iteration, while the gray line represents a smoothed trend obtained using a moving average to highlight the overall evolution of the acquisition-function values. Higher acquisition values indicate regions where the surrogate model predicts greater potential improvement or higher uncertainty, whereas values approaching zero suggest limited expected improvement. The shaded region marks the late optimization stage, where acquisition values remain consistently close to zero, indicating that the parameter space has been largely explored and the optimization primarily performs local refinement around the optimal region.

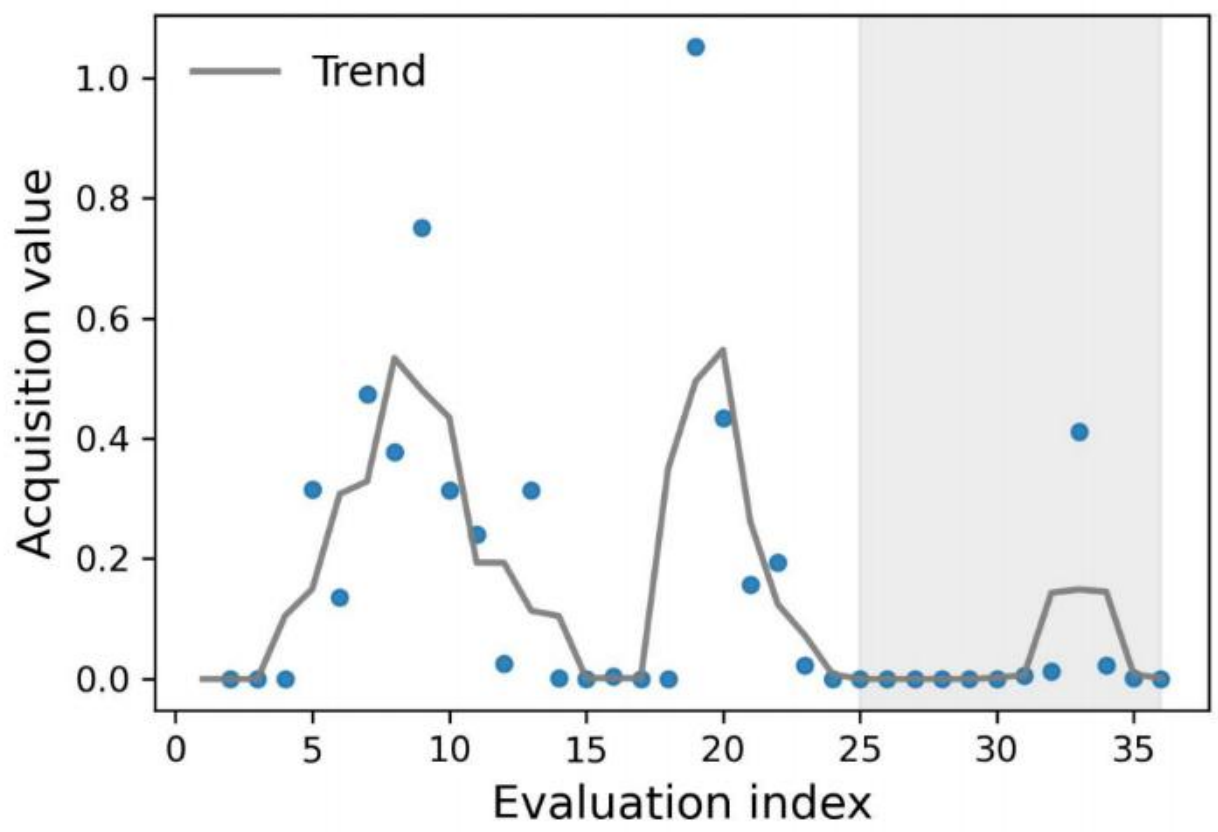

Figure S2. Evolution of acquisition-function values at sampled points during Bayesian optimization.

To further assess the influence of surrogate-model selection on BO performance, we additionally evaluated isotropic Matérn-5/2 Gaussian process (GP) and automatic relevance determination (ARD) Matérn-5/2 GP under the same conditions used for the RF optimization in the main text. Both GP variants used identical initial samples, parameter bounds, EI acquisition function, candidate-generation strategy, simulation setup, and evaluation budget, with only the surrogate model changed. The isotropic GP used a shared length scale across all input dimensions, whereas the ARD GP learned independent length scales for individual dimensions.

Figure S3 shows the BO-guided optimization trajectories after initialization. The isotropic GP exhibited a slower decrease in the best-so-far error and remained in a relatively high-error regime for a longer portion of the search. In contrast, the ARD GP entered the low-error region more rapidly, reflecting its greater flexibility in capturing dimension-dependent parameter sensitivities. Nevertheless, the isotropic and ARD GPs still produced 7 and 4 morphology-penalty evaluations, respectively. Using CCD error > 2.5 nm as the criterion for high-error evaluations, the isotropic GP and ARD GP generated 33 and 20 such evaluations, respectively, compared with 16 for RF in the main text, for which no morphology-penalty evaluations occurred. Thus, under the same simulation budget, RF concentrated a larger fraction of evaluations in the low-error feasible region.

In terms of final calibration accuracy, the ARD GP reached a minimum CCD error of 1.18 nm, close to the 1.28 nm obtained with RF, although the former was slightly lower. Overall, RF achieved calibration accuracy comparable to that of the ARD GP while requiring fewer high-error and defect-related evaluations, indicating higher overall search efficiency and further supporting its use as the BO surrogate in this work.

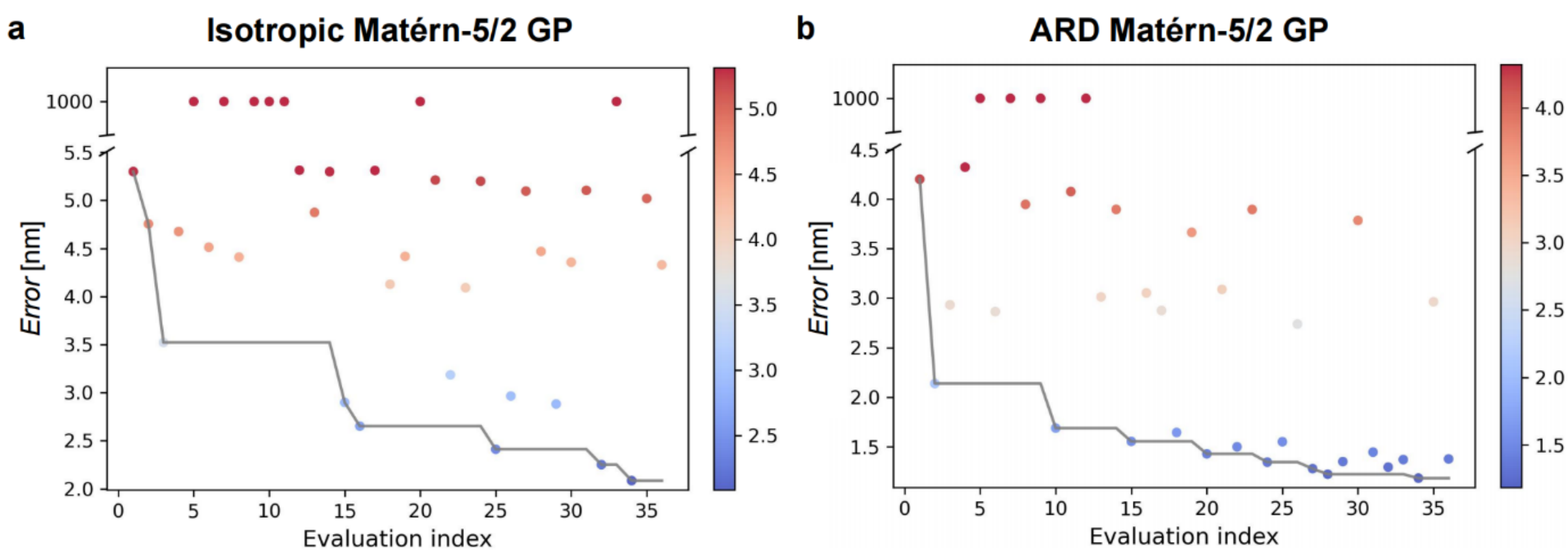

Figure S3. BO optimization trajectories obtained using (a) isotropic Matérn-5/2 GP and (b) ARD Matérn-5/2 GP. Scatter points denote the objective values of individual BO-guided evaluations, and the gray step lines represent the corresponding best-so-far errors. Initial samples are excluded.

5. Convolutional neural network (CNN) details

To eliminate reliance on manual morphology inspection, we develop a data-driven, convolutional neural network (CNN)-based screening module for rapid classification of simulated morphologies.[S10] Using the OK model, we performed systematic 3D simulations across a range of template dimensions and collected 1,800 two-dimensional (2D) cross-sectional composition (density-field) maps extracted from the 3D outputs. The optimization targets parameter sets that yield the experimentally observed, perfect, vertically continuous double-hole morphology. Accordingly, morphology screening is posed as a binary classification task. The dataset comprised 987 defective and 813 perfect samples, with representative examples shown in **Figure S4**. In contrast to the perfect class, which exhibits relatively consistent morphology, the defective class spans diverse failure modes with substantial structural variability. To mitigate bias arising from class imbalance, we oversampled the minority defective subclasses in the training set, such as cylinder breakage and non-convergence cases, by replication and expanded the perfect class by the same amount to maintain an overall balanced distribution. The final dataset contained 1,317 defective and 1,279 perfect samples, which were randomly split into training, validation, and test sets in an 8:1:1 ratio. All samples were preprocessed to ensure a consistent input format across template dimensions. Because the simulations were performed under confined boundary conditions, the density fields showed non-periodic variations near the template boundaries. To avoid distorting these boundary features, we applied symmetric cropping followed by isotropic resizing to a fixed resolution of 128 × 128. The density-field values were then linearly

normalized to [0, 1] and standardized to zero mean, with Gaussian noise added to enhance model robustness.

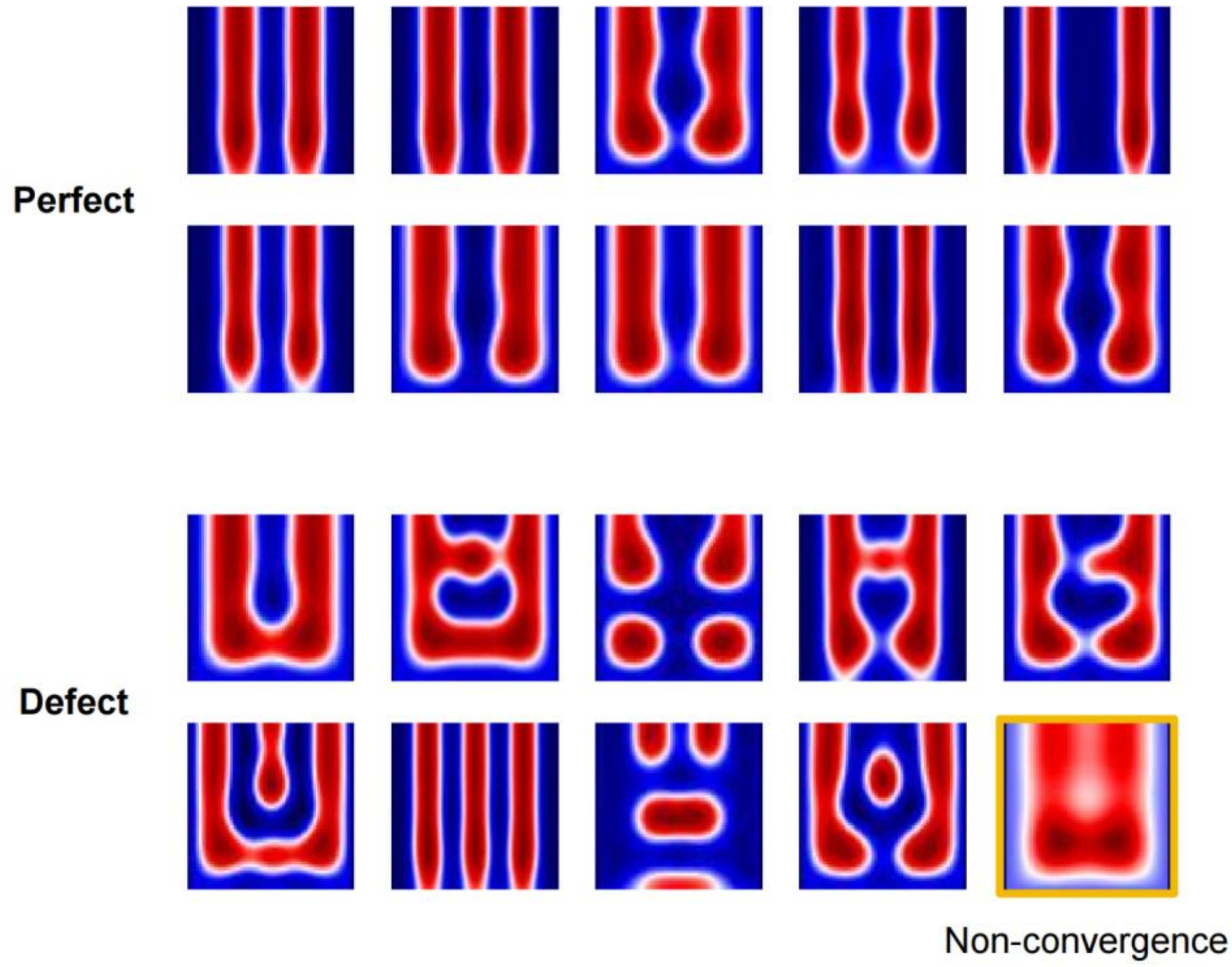

Figure S4. Representative simulation morphologies of perfect, defective, and non-converged samples.

For this binary classification task, normalized and standardized single-channel density-field arrays of size 128 × 128 were used as input and processed by a multilayer convolutional feature extractor followed by a fully connected classification head. The convolutional blocks progressively capture morphological features from local to global scales; each convolution is followed by batch normalization (BN) and a LeakyReLU activation ($\alpha = 0.1$) to improve training stability and enhance nonlinear representation. After the second convolutional block, max pooling and dropout ($p = 0.25$) are applied to reduce overfitting while retaining critical morphological details. Global average pooling is then used to reduce the number of trainable parameters and to improve translational invariance. The resulting features are flattened and passed to a fully connected layer that outputs a single logit, which is converted to a defect probability via a sigmoid function. Since misclassifying defective morphologies as perfect is more detrimental in our workflow, training employs a class-weighted binary cross-entropy loss to emphasize defect recognition, improving recall and reducing false negatives. In addition, the decision threshold is tuned on the validation set to maximize the F1 score while maintaining recall. Model training was performed using the Adam optimizer (learning rate = 0.001, weight decay = 0.0001) with a batch size of 64 for 20 epochs, and early stopping was

enabled based on the validation loss to prevent overfitting. Training, validation, and testing were implemented in PyTorch and performed on a single NVIDIA GeForce RTX 4090 graphics processing unit (GPU).

Under the above training configuration, a lightweight 2D-CNN with three convolutional layers was first constructed as a baseline model for training and evaluation, with the corresponding learning curves shown in **Figure S5a**. At convergence, the model achieved training and validation accuracies of 84.70% and 84.85%, respectively, with corresponding loss values of 0.45 and 0.50. The close agreement between training and validation performance indicates that the baseline network is capable of stably capturing the dominant discriminative features in the density fields and exhibits a reasonable level of generalization. However, noticeable fluctuations remained in both the loss and accuracy curves, with sporadic iterations showing relatively large errors. This behavior suggested that the training stability and robustness could be further improved, motivating subsequent optimization of the network architecture to enhance classification accuracy and screening reliability.

We increased the network depth from three to four convolutional blocks while keeping the training protocol unchanged, aiming to strengthen feature representation and improve classification performance. The training results of the upgraded model are shown in **Figure S5b**. Compared with the three-layer baseline, the four-layer network exhibits a lower loss, reaching training and validation losses of 0.154 and 0.126 at convergence. The corresponding accuracies increased to 95.76% and 94.96%, and the overall accuracy on the independent test set was 96.56%. These improvements indicate that the additional convolutional depth enables more effective extraction of defect-relevant discriminative features from the density fields, leading to enhanced fitting capability and generalization. To further assess classification reliability, the confusion matrix for the test set is provided in **Figure S5c**. The results confirmed robust separation between defective and perfect morphologies, with no defective samples misclassified as perfect, demonstrating stable generalization and reliable defect screening. This level of performance satisfies the accuracy requirements for automated morphology screening and feedback when coupled with the BO module. Accordingly, the four-layer CNN was selected as the morphology classifier in the BO-CNN framework to enable automated screening and feedback updates, establishing a closed-loop workflow that links simulation, defect identification, and parameter optimization.

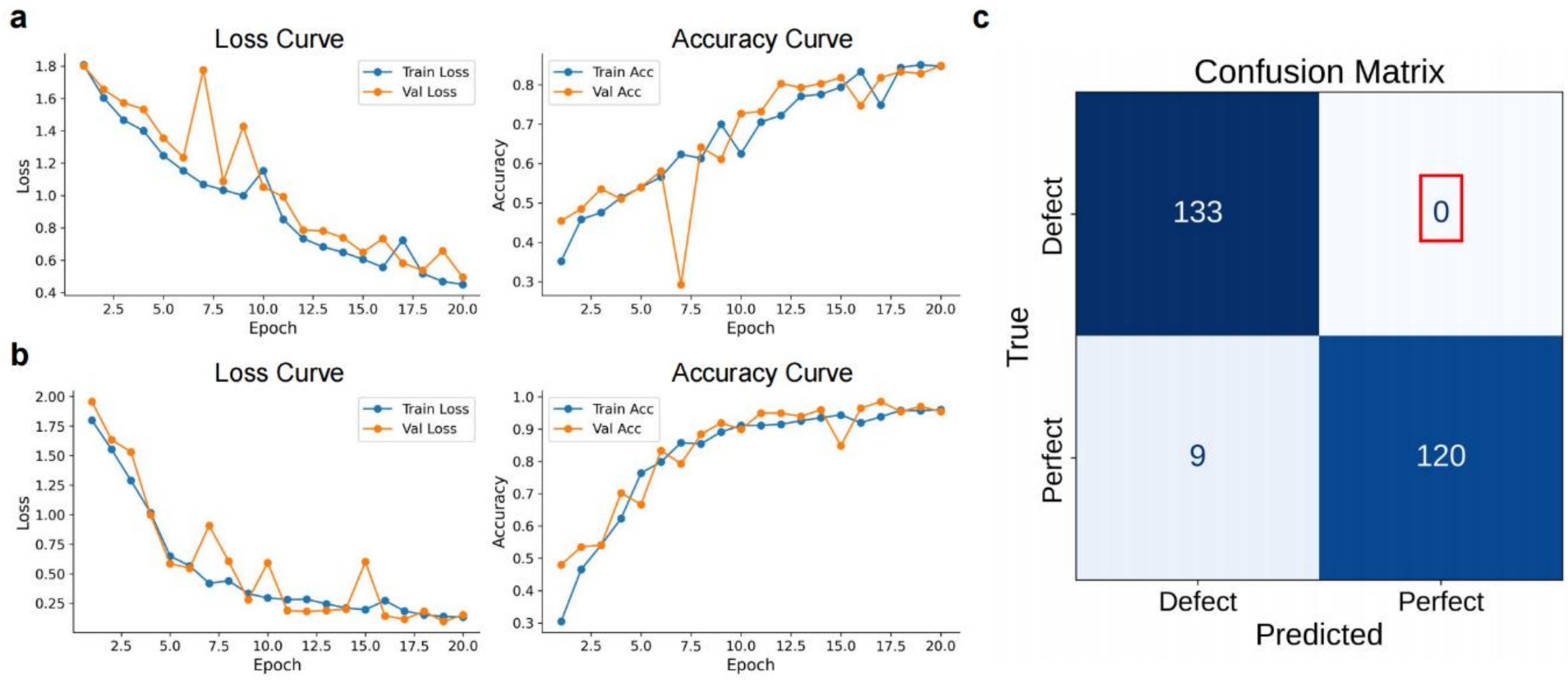

Figure S5. Training and performance evaluation of the CNN models. (a) Loss and accuracy curves of the 3-layer CNN. (b) Loss and accuracy curves of the 4-layer CNN. (c) Confusion matrix of the 4-layer CNN on the test set.

6. Multimodal large language model (LLM)-based screening details

To evaluate the capability of multimodal large language models (LLMs) to classify DSA morphologies under different viewing orientations and image modalities, two binary datasets of double-hole DSA morphologies were constructed. Four representative multimodal LLMs, namely Qwen3.6-Plus (Alibaba Cloud), GLM-5V-Turbo (Z.AI), GPT-5.4 (OpenAI), and Gemini 3.1 Pro Preview (Google), were evaluated in parallel to reduce model-specific bias. The exact application programming interface (API) model identifiers used in this study were qwen3.6-plus, glm-5v-turbo, gpt-5.4, and gemini-3.1-pro-preview, respectively.

Figure S6a presents the side-view grayscale image dataset, whereas **Figure S6b** presents the top-view color image dataset. Each dataset was divided into a training set and a test set. The training set was used to provide few-shot examples in the prompt and contained four defective images and four perfect images. The test set was used for performance evaluation and contained ten defective images and ten perfect images. The side-view grayscale images were used to evaluate the through-thickness continuity of the target double-cylinder morphology and to identify continuity-disrupting defects, including bridging and breakage. The top-view color images primarily reveal the 2D topology of the hole structures, including the number of holes, spatial separation, circular integrity, and defect features such as extra or missing holes.

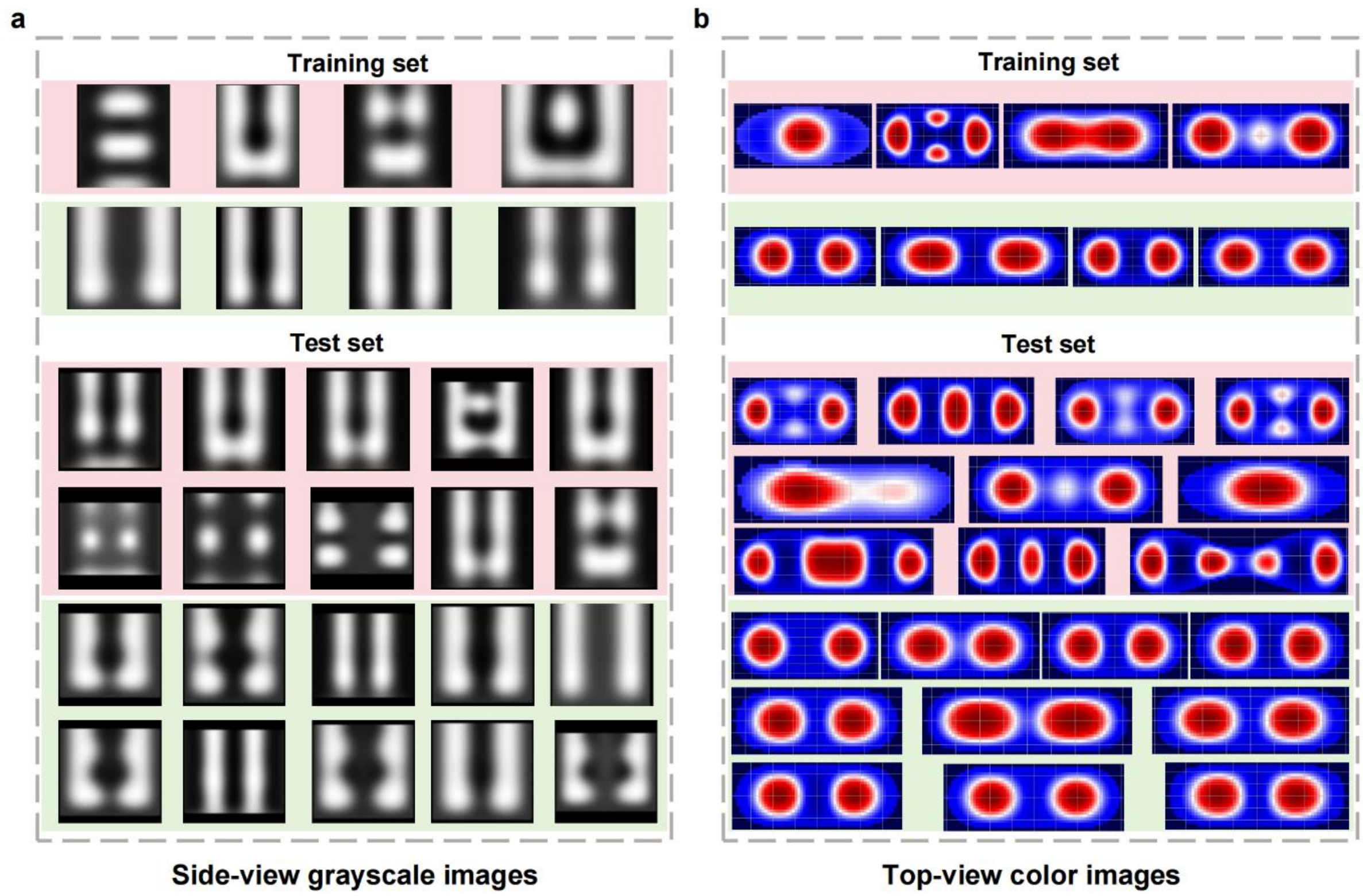

Figure S6. Datasets used for LLM-assisted DSA morphology classification. (a) Side-view grayscale images. (b) Top-view color images.

As shown in **Figure S7**, a hierarchical prompt-engineering strategy was designed for the side-view grayscale images to constrain the LLM-based classification process. Prompt A defined the basic zero-shot classification task by specifying the model role, analysis sequence, and structured output format. Prompt B extended this setting by introducing global morphology-based criteria, with explicit definitions of perfect and defect morphologies. Prompt C further incorporated labeled reference images with objective descriptions, enabling few-shot morphology comparison. Because defect rejection is prioritized in the morphology-screening task, a conservative defect criterion was adopted in the prompts. Specifically, images showing bridging, breakage, residue, discontinuity, pronounced asymmetry, or ambiguous morphology were preferentially classified as defective. This conservative rule improves defect-screening capability, although it may also classify some borderline perfect samples as defective.

Shared task instructions for Prompts A-C

You are an expert evaluator for Directed Self-Assembly (DSA) simulation morphology.
Your task is to classify a simulation image as either perfect or defect based only on visible morphology in the image.
Do not use filename, folder name, prior label, or any external information.

Analyze the image in the following order:
1. Identify the main domains and the overall topology.
2. Check **whether the expected domains are clearly present, separated, continuous, and complete**.
3. Inspect **visible defect evidence, including bridge, break, residue, asymmetry, distortion, incomplete domain formation, or topology mismatch**.
4. Make the final classification based only on directly visible image evidence.
Return JSON only and do not output any additional text.

Global morphology rules for Prompts B-C

Global Rules for Pure Image-Based DSA Classification

Task
Classify each input DSA simulation image as either perfect or defect based only on visible morphology in the image.
Scope
This rule set is designed for double-hole DSA images under a pure image-based evaluation setting.

Classification Criteria
Perfect
An image is classified as perfect when two main domains are clearly present, separated, continuous, complete, and approximately symmetric, with no obvious bridge, break, extra island or residue, or severe distortion.
Defect
An image is classified as defect when any obvious abnormal morphology is visible, including bridge, break, residue, asymmetry, distortion, incomplete_domain, or other clear deviation from a normal double-hole pattern.

Decision Rule
- Use only visible image evidence.
- Do not use filename, folder name, prior label, or external information.
- Classify as perfect only when no obvious defect evidence is observed.
- **If any clear abnormality is visible, classify as defect.**
- **If the morphology is ambiguous or not confidently perfect, prefer defect.**

Few-shot reference examples for Prompt C

image_name,label,defect_type,objective_description

defect/1.png,defect,"bridge;residue","The two main domains are connected by a bridge, with additional residual structure between the two cylinders."
defect/2.png,defect,"bridge","The two main domains are connected by a clear bridge."
defect/3.png,defect,"bridge;break","The two domains are connected, and at least one domain is broken or not fully continuous."
defect/4.png,defect,"break;incomplete_domain","Only one cylinder is visible, and the expected double-domain structure is not fully formed."
defect/5.png,defect,"break","Two main domains are present, but one or both are broken and not continuous."
defect/6.png,defect,"break","Two cylinders are visible, but the morphology is not clearly continuous and does not form a standard complete cylindrical pattern."
defect/7.png,defect,"other","Three cylinder-like domains are visible instead of the expected two-domain pattern."
perfect/1-4.pngs,perfect,"none","Two main domains are clearly separated, continuous, complete, and approximately symmetric."

Figure S7. Hierarchical prompt design for side-view grayscale DSA morphology screening.

For the top-view color images shown in **Figure S8**, the prompts were designed to emphasize plan-view geometric morphology. Compared with the side-view prompts, the top-view prompts focused on the target pattern of two well-separated, complete, size-matched, and approximately symmetric circular holes, while explicitly identifying defects such as extra holes, missing holes, bridging, and merged structures. The hierarchical structure of prompts A, B, and C was kept consistent with that used for the side-view images, allowing direct comparison among zero-shot, rule-guided, and few-shot settings. A stringent defect criterion was adopted: only images with two clearly resolved and geometrically regular holes were classified as perfect, whereas any visible abnormality or morphological ambiguity was

assigned to the defect class. This conservative strategy prioritizes defect rejection and minimizes missed defect cases in the preliminary screening workflow.

Shared task instructions for Prompts A-C

You are an expert evaluator for Directed Self-Assembly (DSA) simulation morphology.
Your task is to classify a simulation image as either perfect or defect based only on visible morphology in the image.
Do not use filename, folder name, prior label, or any external information.
Analyze the image in the following order:
1. Identify the **main circular** domains and **the overall top-view morphology**.
2. Check whether **exactly two main circular domains are clearly present, well separated, complete, and approximately symmetric**.
3. Inspect visible defect evidence, including bridge or merged domains, missing or incomplete domains, extra islands or residue, severe asymmetry, irregular shape, strong size mismatch, distortion, off-center distribution, unclear separation, or topology mismatch.
4. Make the final classification based only on directly visible image evidence.
Return JSON only and do not output any additional text.

Global morphology rules for Prompts B-C

Global Rules for Pure Image-Based DSA Classification

Task
Classify each input DSA simulation image as either perfect or defect based only on visible morphology in the image.
Scope
This rule set is designed for double-hole DSA top-view images under a pure image-based evaluation setting.

Classification Criteria
Perfect
An image is classified as perfect when two main circular domains are clearly present, well separated, complete, and approximately symmetric. The two domains should have similar size, regular round shape, and balanced spatial distribution, with no obvious bridge, residue, extra island, missing region, severe deformation, or abnormal connection.
Defect
An image is classified as defect when any obvious abnormal morphology is visible, including bridge, merged domains, missing or incomplete domains, extra islands or residue, severe asymmetry, irregular shape, strong size mismatch, distortion, off-center distribution, unclear separation, or other clear deviation from a normal double-hole top-view pattern.

Decision Rule
- Use only visible image evidence.
- Do not use filename, folder name, prior label, or external information.
- Classify as perfect only when two separated, symmetric, and approximately circular domains are clearly observed.
- **If any clear abnormality is visible, classify as defect.**
- **If the morphology is ambiguous or not confidently perfect, prefer defect.**

Few-shot reference examples for Prompt C

image_name,label,defect_type,objective_description

defect/1.png,defect,"extra_domain","Three hole-like domains are visible instead of the expected two-hole pattern, with an additional small hole located between the two main holes."
defect/2.png,defect,"bridge","The two main holes are connected or merged together, and the expected clear separation between two circular domains is lost."
defect/3.png,defect,"missing_domain","Only one hole is visible, and the expected double-hole structure is not fully formed."
defect/4.png,defect,"extra_domains","Four hole-like domains are visible instead of the expected two-hole pattern, with two additional small holes vertically arranged between the main domains."
perfect/1-4.pngs,perfect,"none","Two circular hole domains are clearly visible, well separated, approximately equal in size, and symmetrically distributed."

Figure S8. Hierarchical prompt design for top-view color DSA morphology screening.

To facilitate automated parsing and statistical analysis, the LLMs were required to return classification results in a strict JavaScript Object Notation (JSON) format. As shown in **Figure S9**, both side-view and top-view tasks used a unified output schema containing the core fields label, confidence, defect_type, morphology_features, rule_judgment, reason, and keep_for_bo. The label field records the perfect or defective classification, the confidence field reports the model confidence, and the defect_type field specifies the identified defect category. The morphology_features and reason fields provide interpretable morphology-based

evidence, whereas the rule_judgment field converts the decision criteria into Boolean variables for automated programmatic reading. The keep_for_bo field is designed as a binary eligibility flag for potential integration into a downstream BO-based parameter-optimization workflow. For side-view images, the output focused on the separation and continuity of the double-domain structure. For top-view images, it emphasized the number of circular holes, size similarity, and spatial symmetry.

a

"label": "perfect",
"confidence": 0.93,
"defect_type": "none",
"morphology_features": [
"two main domains are clearly present",
"domains are separated and continuous",
"overall morphology is approximately symmetric"
],
"rule_judgement": {
"two_main_domains_present": true,
"clearly_separated": true,
"domains_complete": true,
"no_bridge": true,
"no_extra_residue": true
},
"reason": "Two main domains are clearly separated, continuous, and complete, with no obvious defect evidence.",
"keep_for_bo": true

Side-view output

b

"label": "perfect",
"confidence": 0.93,
"defect_type": "none",
"morphology_features": [
"two circular domains are clearly present",
"the two domains are well separated",
"the domains have similar size and symmetric distribution"
],
"rule_judgement": {
"two_circular_domains_present": true,
"clearly_separated": true,
"similar_size": true,
"symmetric_distribution": true,
"no_bridge": true,
"no_extra_or_missing_domain": true
},
"reason": "Two circular domains are clearly separated, similarly sized, and approximately symmetrically distributed, with no obvious bridge, extra domain, or missing domain.",
"keep_for_bo": true

Top-view output

Figure S9. Representative structured outputs of LLM-assisted DSA morphology classification. (a) Example JSON output for side-view grayscale image classification. (b) Example JSON output for top-view color image classification.

Figure S10 summarizes the inference cost of different LLMs for the side-view classification task. Among the four models, Qwen3.6-Plus showed the lowest unit token cost and total cost, indicating the highest cost-effectiveness. In contrast, Gemini 3.1 Pro Preview incurred the highest total cost, mainly because its image inputs were converted into a substantially larger number of tokens than those of the other models, resulting in higher input-token consumption under the same task setting. Although GPT-5.4 had the highest unit token cost, its lower overall token usage led to a lower total cost than that of Gemini 3.1 Pro Preview. Notably, despite the relatively high cost of Gemini 3.1 Pro Preview, the expense of a complete classification task remained within an acceptable range and did not limit its use in small-scale validation or model-comparison experiments. The cost trend for the top-view

classification task was similar to that of the side-view task and is therefore not shown separately.

a

Qwen3.6-Plus					
Prompt	Input token	Output token	RMB/M token		Cost per image
			Input	Onput	
A	120	173.86			0.00233
B	131	168.67			0.00229
C-2	144	169.12	2	12	0.00232
C-4	154	174.14			0.00240
C-8	176	176.95			0.00248
Total Cost = RMB 1.182					

b

GLM-5V-Turbo					
Prompt	Input token	Output token	RMB/M token		Cost per image
			Input	Onput	
A	70.45	262.06			0.006
B	81.60	280.45			0.006
C-2	89.45	253.97	5	22	0.006
C-4	94.95	211.53			0.005
C-8	106.8	259.81			0.006
Total Cost = RMB 3.01					

c

GPT-5.4					
Prompt	Input token	Output token	RMB/M token		Cost per image
			Input	Onput	
A	112	166.60			0.021
B	123	167.91			0.021
C-2	136	172.83	18.8	112.5	0.022
C-4	146	171.26			0.022
C-8	166	169.36			0.022
Total Cost = RMB 10.8					

d

Gemini 3.1 Pro Preview					
Prompt	Input token	Output token	RMB/M token		Cost per image
			Input	Onput	
A	1147	400.81			0.053
B	1159	390.61			0.053
C-2	1274	418.16	15	90	0.057
C-4	1387	334.54			0.051
C-8	1614	339.93			0.055
Total Cost = RMB 26.83					

Figure S10. Cost analysis of side-view DSA morphology classification across different LLMs. (a) Qwen3.6-Plus, (b) GLM-5V-Turbo, (c) GPT-5.4, and (d) Gemini 3.1 Pro Preview under different prompt settings (A, B, C-2, C-4, and C-8). RMB denotes Chinese renminbi. Token numbers and cost values in each row denote the average consumption for classifying one image. The section-level total cost is calculated by summing the costs over 20 test images and 5 repeated runs.

Figure S11 summarizes the individual metric performance of four LLMs under different prompt settings for the side-view grayscale classification task. Overall, the classification accuracy varied only moderately across prompt settings, indicating limited prompt-dependent changes in global classification performance. In contrast, $\mathrm{recall}_{\mathrm{defect}}$ and $\mathrm{recall}_{\mathrm{perfect}}$ showed stronger model-specific sensitivity to prompt engineering. Gemini 3.1 Pro Preview maintained consistently high $\mathrm{recall}_{\mathrm{defect}}$ with limited variation across prompts, but showed relatively low $\mathrm{recall}_{\mathrm{perfect}}$, suggesting a stringent decision strategy that tended to classify borderline perfect samples as defective. For GPT-5.4, $\mathrm{recall}_{\mathrm{defect}}$ increased markedly after more explicit morphology rules and reference examples were introduced and remained high

from Prompt C-2 onward, whereas $recall_{perfect}$ remained comparatively low. Qwen3.6-Plus achieved a more balanced trade-off between $recall_{defect}$ and $recall_{perfect}$, whereas GLM-5V-Turbo exhibited larger metric fluctuations across prompt settings, indicating lower classification stability. Because the LLMs are used here as a preliminary defect-screening tool, $recall_{defect}$ is the most critical metric. Accordingly, Gemini 3.1 Pro Preview and GPT-5.4 are better suited for defect-sensitive screening in this task. In terms of system usability, all four LLMs showed label-reason consistency, indicating that their textual explanations were generally aligned with the assigned labels. The average inference time was also on the order of seconds, suggesting an acceptable time cost for practical screening.

As a supervised-learning baseline, the trained CNN classifier was evaluated on the same side-view test set. **Figure S12** shows the resulting confusion matrix. The CNN achieved high classification performance, with an accuracy of 0.95, a $recall_{defect}$ of 1.00, a $recall_{perfect}$ of 0.90, and an average inference time of only 0.01 s per image.

Overall, the CNN provided higher accuracy and inference efficiency within the fixed training distribution, making it suitable for rapid, fine-grained classification of known datasets. However, its reliance on labeled training data limits its flexibility, as new template geometries, defect modes, or evaluation criteria would typically require additional annotation and retraining. In contrast, LLMs can adapt the decision criteria through textual rules and a few reference examples without further training. In this study, the conservative prompt design yielded stable $recall_{defect}$ performance, albeit at the expense of reduced $recall_{perfect}$. Thus, LLMs can serve as an interpretable and transferable preliminary evaluation module that enhances the adaptability and generalization potential of the automated screening framework.

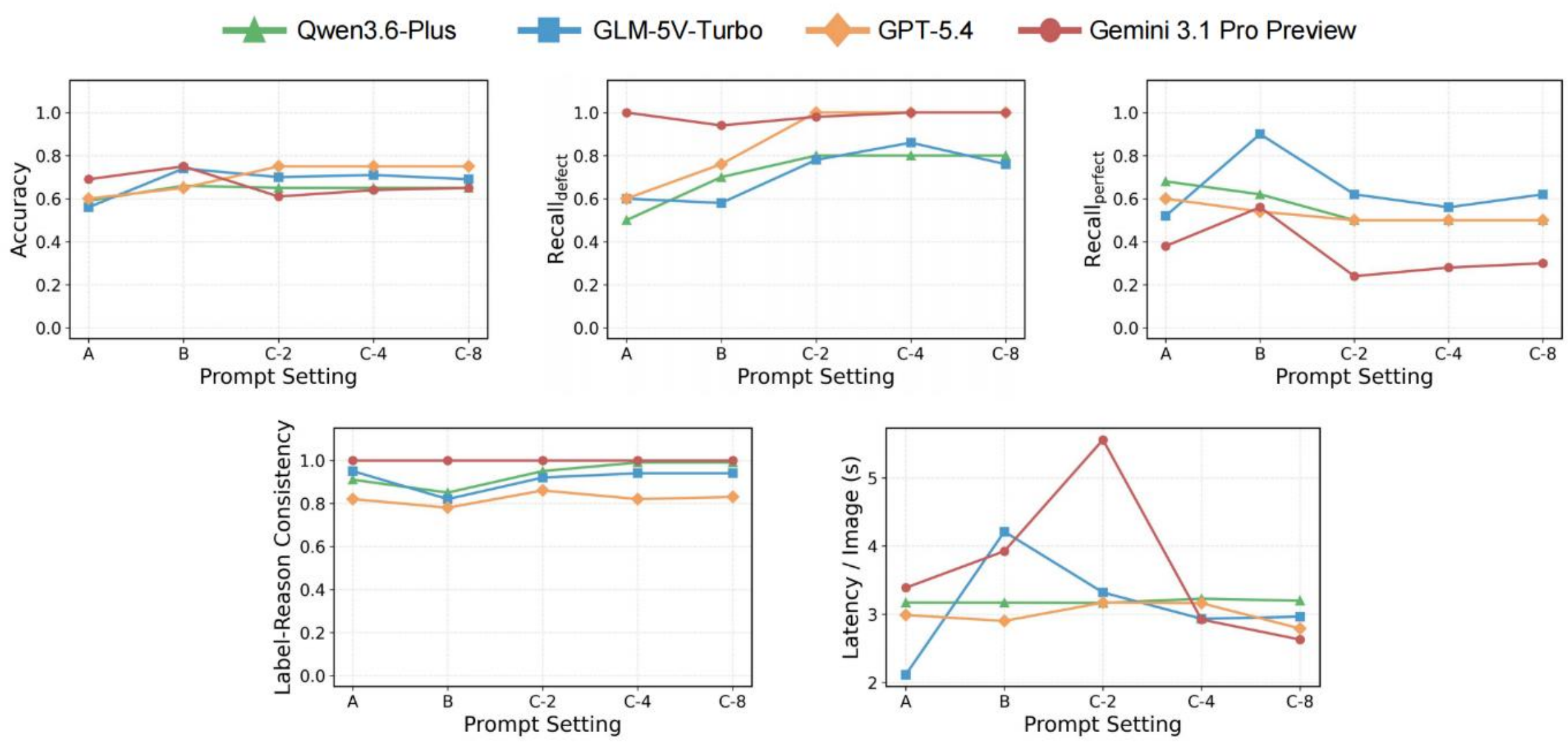

Figure S11. Individual-metric evaluation of side-view grayscale DSA morphology classification across different LLMs.

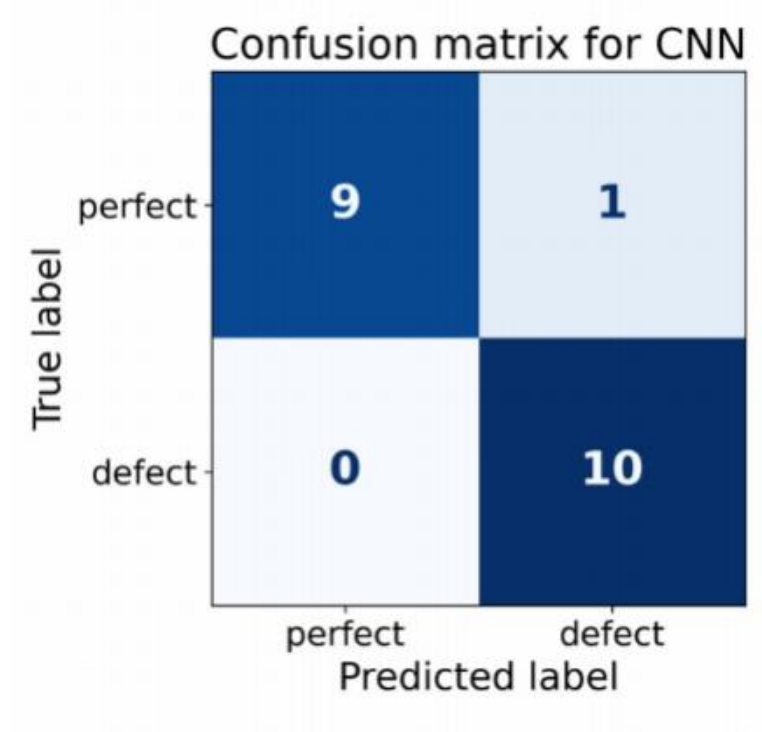

Figure S12. Confusion matrix of the CNN classifier for side-view grayscale DSA morphology classification.

Figure S13 summarizes the individual metric performance of the four LLMs for the top-view color classification task. The top-view results were markedly better than those obtained from side-view grayscale images. Except for GLM-5V-Turbo, which showed pronounced fluctuations under several prompt settings, most models achieved high accuracy, $\text{recall}_{\text{defect}}$, and $\text{recall}_{\text{perfect}}$ values that approached 1.00 across most prompts. Among them, Gemini 3.1 Pro Preview delivered the most stable and consistently highest performance across all metrics. This improvement suggests that top-view color images are more compatible with LLM-based visual interpretation than side-view grayscale images, because they provide stronger visual contrast and more explicit topological cues. These features enable more reliable identification of bridging, extra holes, missing holes, and incorrect hole numbers, whereas side-view defects are often subtle and embedded in weak grayscale variations. The average inference time for all models also remained on the order of seconds, indicating an acceptable time cost for practical screening.

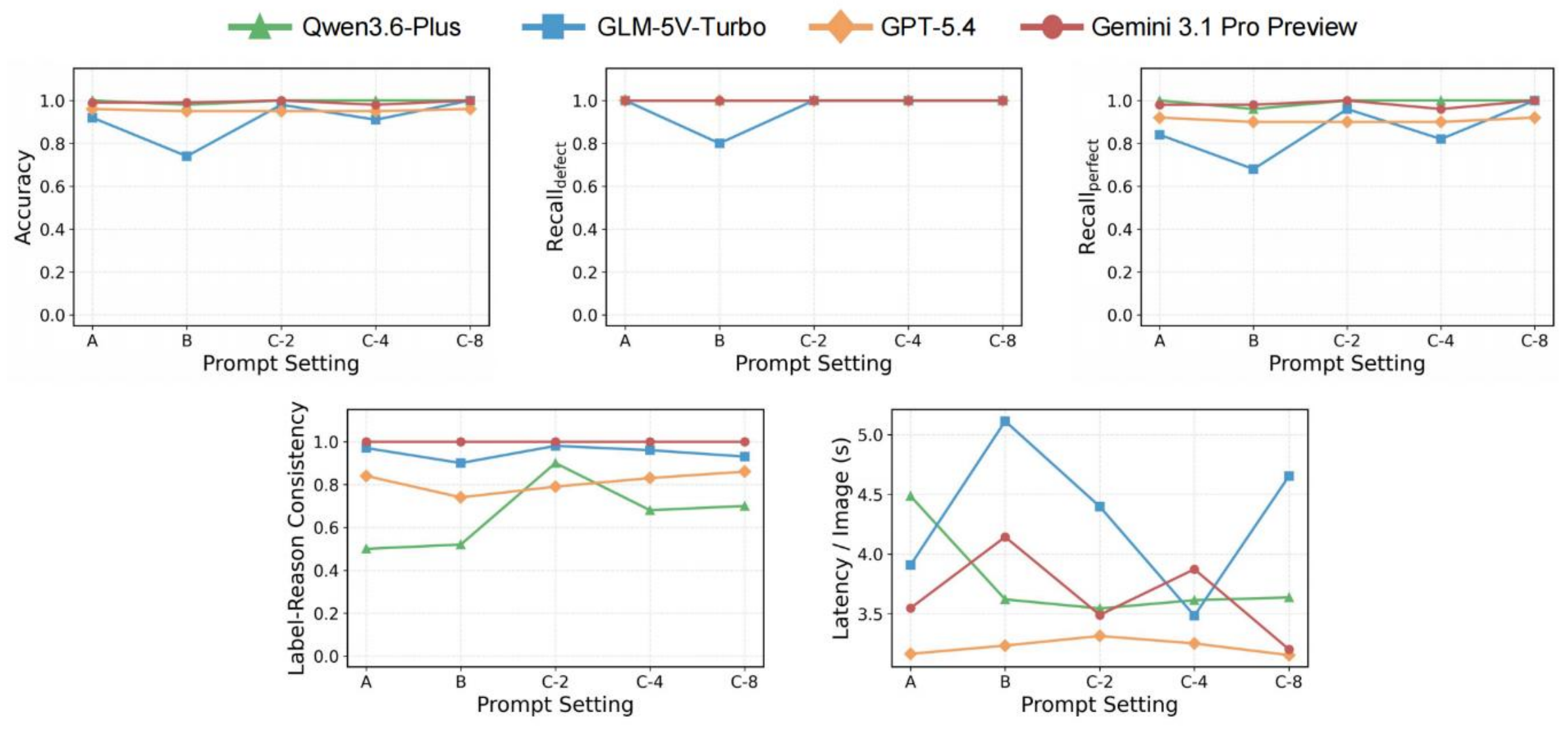

Figure S13. Individual-metric evaluation of top-view color DSA morphology classification across different LLMs.

Overall, LLMs provide rapid adaptability to new image modalities and view-dependent morphological representations without additional task-specific fine-tuning, while showing particular strength in morphology classification tasks characterized by clear topological features and strong regional contrast.

To comprehensively evaluate the overall performance of different LLMs and prompt settings in DSA morphology classification, a weighted multi-metric scoring scheme was used. The evaluation metrics included accuracy, $\mathrm{recall}_{\mathrm{defect}}$, $\mathrm{recall}_{\mathrm{perfect}}$, label-reason consistency, and average latency per image, covering overall classification performance, defect-screening capability, explanation reliability, and inference efficiency. Accuracy, $\mathrm{recall}_{\mathrm{defect}}$, $\mathrm{recall}_{\mathrm{perfect}}$, and label-reason consistency were treated as positive metrics, for which higher values indicate better performance. Average latency was treated as a negative metric and was converted into a latency score by inverse normalization before scoring.

The metric weights are listed in **Table S2**. This weighting scheme reflects the role of LLMs in morphology screening, where the primary objective is to minimize the misclassification of defect samples as perfect. Accordingly, $\mathrm{recall}_{\mathrm{defect}}$ was assigned the highest weight of 0.35. Accuracy was used to quantify overall classification performance, $\mathrm{recall}_{\mathrm{perfect}}$ was used to assess whether the model was overly conservative in rejecting acceptable morphologies, and label-reason consistency was used to evaluate the agreement between the textual explanation and the assigned label; each of these metrics was assigned a weight of 0.20. Average latency per image reflected the time cost of model invocation. Because the average single-image response time of all LLMs remained within an acceptable

range for the present test scale, latency was not considered a dominant factor affecting model usability and was assigned a lower weight of 0.05.

The overall score was calculated as follows:

$$Score = 100 \times \left(0.35R_{\mathrm{defect}} + 0.20A + 0.20R_{\mathrm{perfect}} + 0.20C + 0.05L_{\mathrm{score}}\right) \quad \textbf{(S8)}$$

where R_{defect}, A, R_{perfect}, C, and L_{score} denote $\mathrm{recall}_{\mathrm{defect}}$, accuracy, $\mathrm{recall}_{\mathrm{perfect}}$, label-reason consistency, and the normalized latency score, respectively.

The latency score was obtained using inverse min-max normalization:

$$L_{\mathrm{score}} = \frac{L_{\max} - L_i}{L_{\max} - L_{\min}} \quad \textbf{(S9)}$$

where L_i is the average single-image inference time for the i-th model-prompt combination, and $L_{\max}$ and $L_{\min}$ are the maximum and minimum latency values among all combinations, respectively. If all combinations had identical latency values, L_{score} is set to 1 for all cases.

Table S2. Weight settings for the comprehensive scoring of LLM classification performance.

Metric	Weight
$\mathrm{Recall}_{\mathrm{defect}}$	0.35
Accuracy	0.20
$\mathrm{Recall}_{\mathrm{perfect}}$	0.20
Label-Reason Consistency	0.20
Latency	0.05

Table S3. Summary of experimental and simulated CCD results for three datasets: the training set (14 samples), the validation set (10 samples), and the prediction set (4 samples).

CDX [nm]	CDY [nm]	CCD_{exp} [nm]	CCD_{sim} [nm]	Error [nm]
		**Training data results**		
54.15	117.01	47.8	48.58	0.78
54.92	120.37	49.3	48.48	0.82
54.38	122.50	50.6	50.37	0.23
55.37	122.96	50.3	50.59	0.29
56.85	119.18	49.2	48.70	0.50
57.70	125.90	51.2	52.00	0.80
57.92	128.23	53.0	52.40	0.60
56.04	128.63	52.9	53.58	0.68
59.98	108.75	44.5	43.49	1.01
58.08	121.91	50.1	50.17	0.07
59.96	127.07	51.8	52.06	0.26
66.52	128.41	50.7	53.00	2.30
66.29	137.13	56.0	58.70	2.70

CDX [nm]	CDY [nm]	CCD_{exp} [nm]	CCD_{sim} [nm]	Error [nm]
75.42	119.22	46.8	49.54	2.74
		**Validation data results**		
53.83	119.01	48.4	46.80	1.60
54.69	118.34	47.8	46.60	1.20
55.50	125.64	51.5	53.80	2.30
56.83	121.45	49.8	50.00	0.20
56.58	124.56	50.9	52.33	1.43
57.10	126.87	52.7	51.80	0.90
59.84	116.20	47.0	48.20	1.20
59.42	128.84	53.4	53.60	0.20
66.99	124.69	49.5	52.38	2.88
74.03	115.21	44.8	46.60	1.80
		**Prediction data results**		
52.04	127.32	50.7	51.99	1.29
52.63	131.65	54.6	55.87	1.27
56.74	115.7	47.2	46.97	0.23
59.51	123.48	49.2	51.87	2.67

CDX and CDY denote the short- and long-axis dimensions of the track-shaped templates, respectively. CCD_{exp} and CCD_{sim} represent the experimental and simulated CCD. Error is their absolute difference.

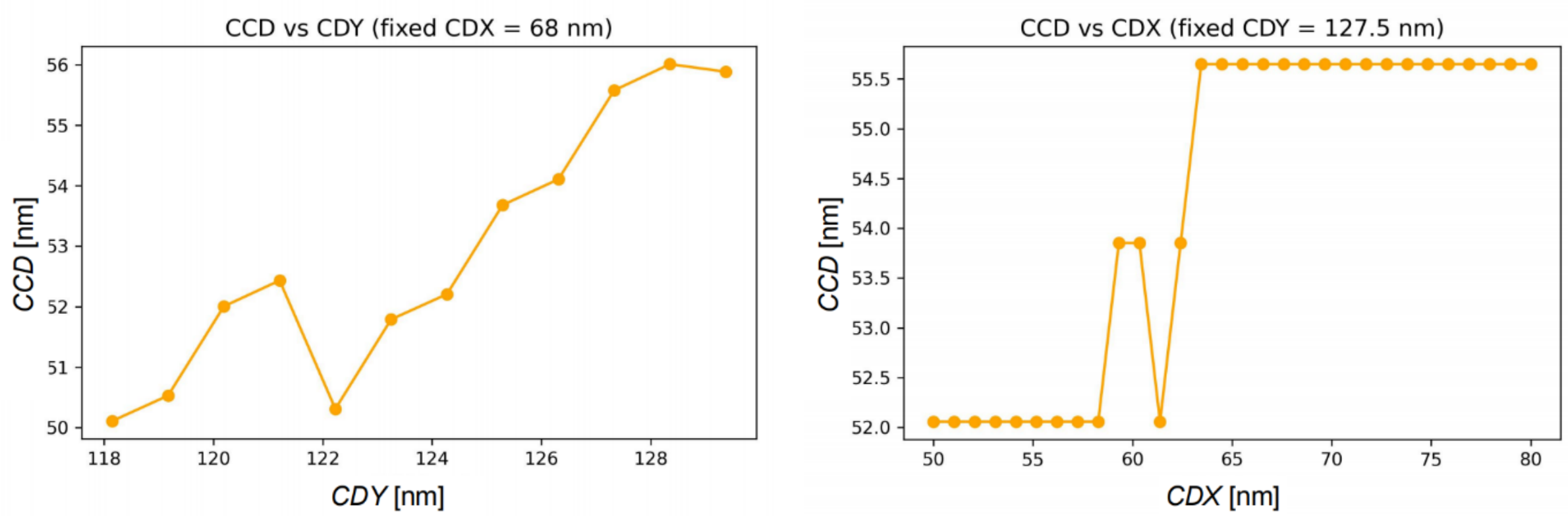

Figure S14. CCD response to variations in CDX and CDY.

7. External validation of the calibrated model using racetrack templates from an independent wafer

To further assess the predictive capability of the calibrated model, 10 racetrack-template samples were selected from a wafer independent of that used in the original dataset. The samples span representative wafer locations, including the center, intermediate, near-edge, and edge regions, and cover different template dimensions, as summarized in **Table S4**.

For all 10 samples, the four calibrated model parameters were kept fixed and directly used for forward simulation. The model achieved a mean absolute CCD error of 0.55 nm, with all deviations below 1.5 nm and a maximum error of 1.31 nm.

Table S4. External validation of the calibrated model using 10 racetrack-template samples from an independent wafer.

CDX [nm]	CDY [nm]	CCD_{exp} [nm]	CCD_{sim} [nm]	Error [nm]
65.17	113.96	48.44	48.81	0.37
61.90	124.28	52.62	52.21	0.41
63.62	120.93	51.44	52.31	0.87
65.47	123.95	52.84	52.07	0.77
68.39	118.29	50.54	50.17	0.37
65.92	129.04	54.98	55.54	0.56
70.81	122.12	52.64	52.06	0.58
73.81	125.51	53.74	53.77	0.03
77.51	128.36	55.74	56.01	0.27
69.78	127.73	54.24	55.55	1.31

CDX and CDY denote the short- and long-axis dimensions of the track-shaped templates, respectively. CCD_{exp} denotes the corrected experimental CCD value, whereas CCD_{sim} denotes the simulated CCD value. Error is their absolute difference.

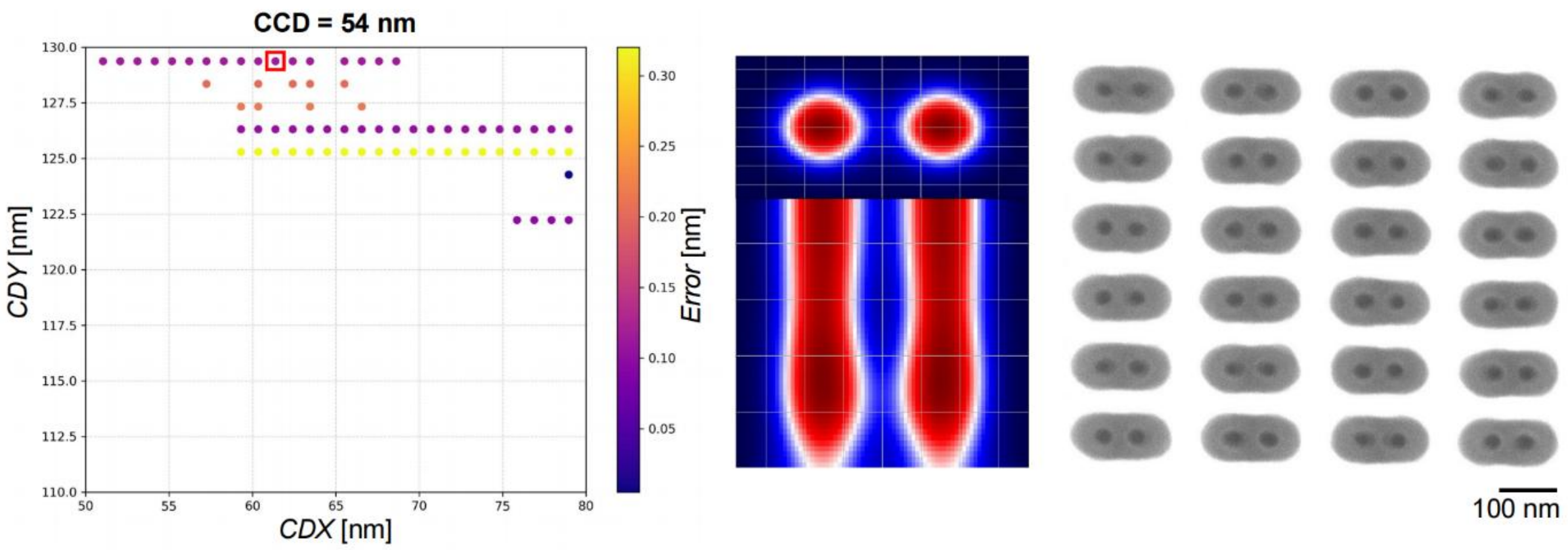

Figure S15. Inverse design window of templates subject to a CCD constraint of 54 ± 1 nm and experimental validation. Scale bars are provided for reference.

8. Robustness analysis of the inverse-design process windows

To evaluate the robustness of the inverse-design process windows with respect to variations in the calibrated parameters, three additional parameter sets were selected from the late-stage low-error BO region. For each parameter set, the feasible regions corresponding to CCD = 52 ± 1 nm and CCD = 54 ± 1 nm were recalculated using the same CDX-CDY template grid and identical inverse-design criteria as those used in the main text. The selected parameter sets were $(\phi_s, \phi_b, \Lambda_s, \Lambda_b) = (0.010, 0.046, 2.45, 1.57)$, $(0.025, 0.066, 4.79, 3.91)$,

and $(0.015, 0.022, 9.30, 3.59)$, representing distinct solutions within the low-error region of the BO search.

As shown in **Figure S16a**, for CCD = 52 ± 1 nm, the three parameter sets yield 114, 111, and 113 feasible template points, respectively, compared with 106 points obtained using the representative best-performing parameter set reported in the main text. For CCD = 54 ± 1 nm, the corresponding numbers are 73, 74, and 69, compared with 76 for the representative parameter set (**Figure S16b**). Despite the differences in calibrated parameters, the resulting feasible regions exhibit similar overall distributions for both CCD targets. In addition, no morphological defects were observed for any of the three additional parameter sets, indicating consistent morphological feasibility across the low-error parameter region.

The red squares in Figure S16 denote the template geometries experimentally validated in the main text. All experimentally validated template points remain within the common intersection of the four feasible regions. The intersection-to-union ratios of the four process windows are 68.25% for CCD = 52 ± 1 nm and 62.07% for CCD = 54 ± 1 nm, confirming substantial overlap among the feasible regions.

These results indicate that parameter variations within the low-error region primarily affect the local boundaries of the design windows, while the principal feasible regions and experimentally validated design points remain stable. The inverse-design process windows therefore exhibit robust behavior with respect to the non-uniqueness of the calibrated parameters.

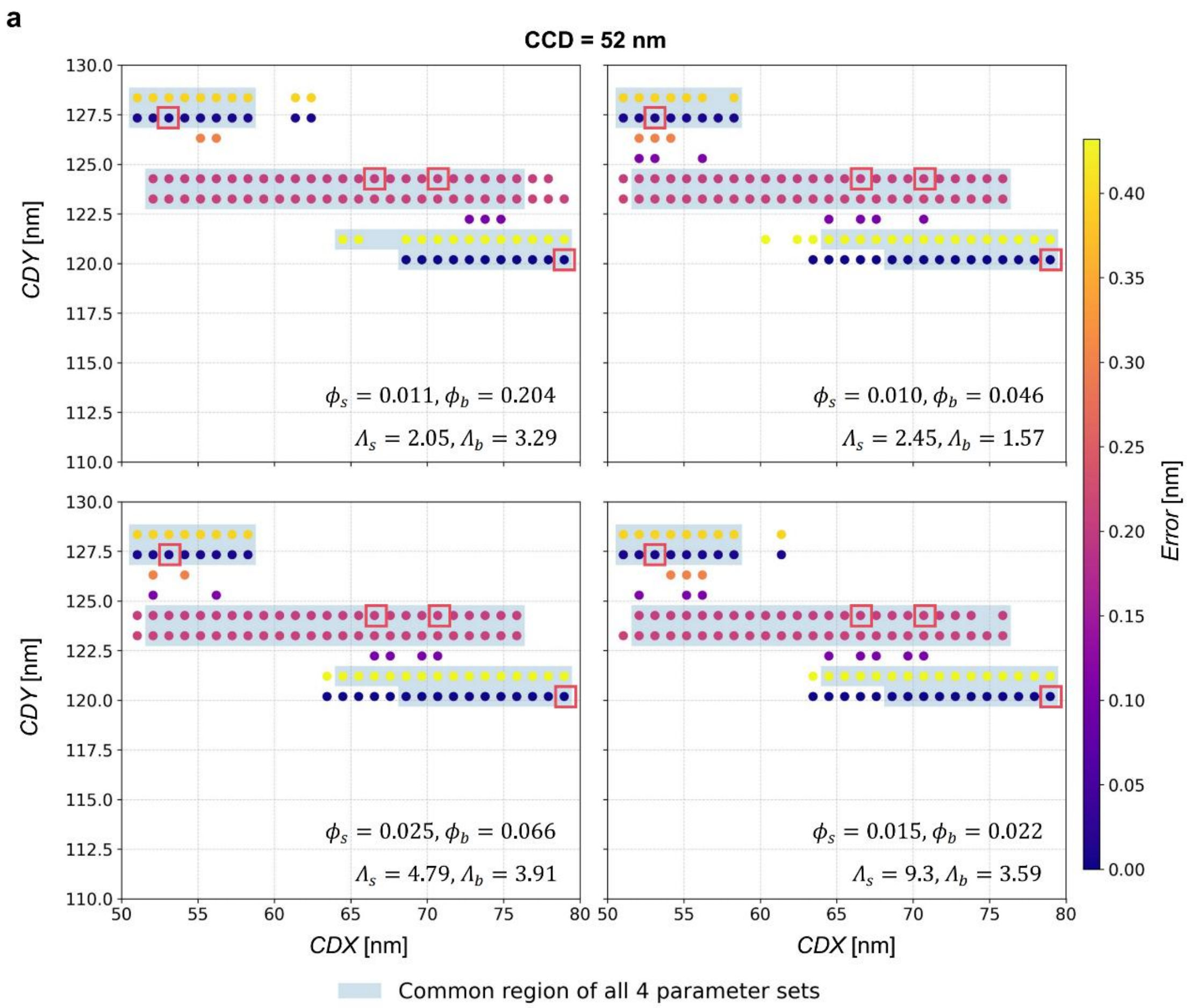
a
CCD = 52 nm
CDY [nm]
CDX [nm]
Error [nm]
$\phi_s = 0.011, \phi_b = 0.204$
$\Lambda_s = 2.05, \Lambda_b = 3.29$
$\phi_s = 0.010, \phi_b = 0.046$
$\Lambda_s = 2.45, \Lambda_b = 1.57$
$\phi_s = 0.025, \phi_b = 0.066$
$\Lambda_s = 4.79, \Lambda_b = 3.91$
$\phi_s = 0.015, \phi_b = 0.022$
$\Lambda_s = 9.3, \Lambda_b = 3.59$
Common region of all 4 parameter sets

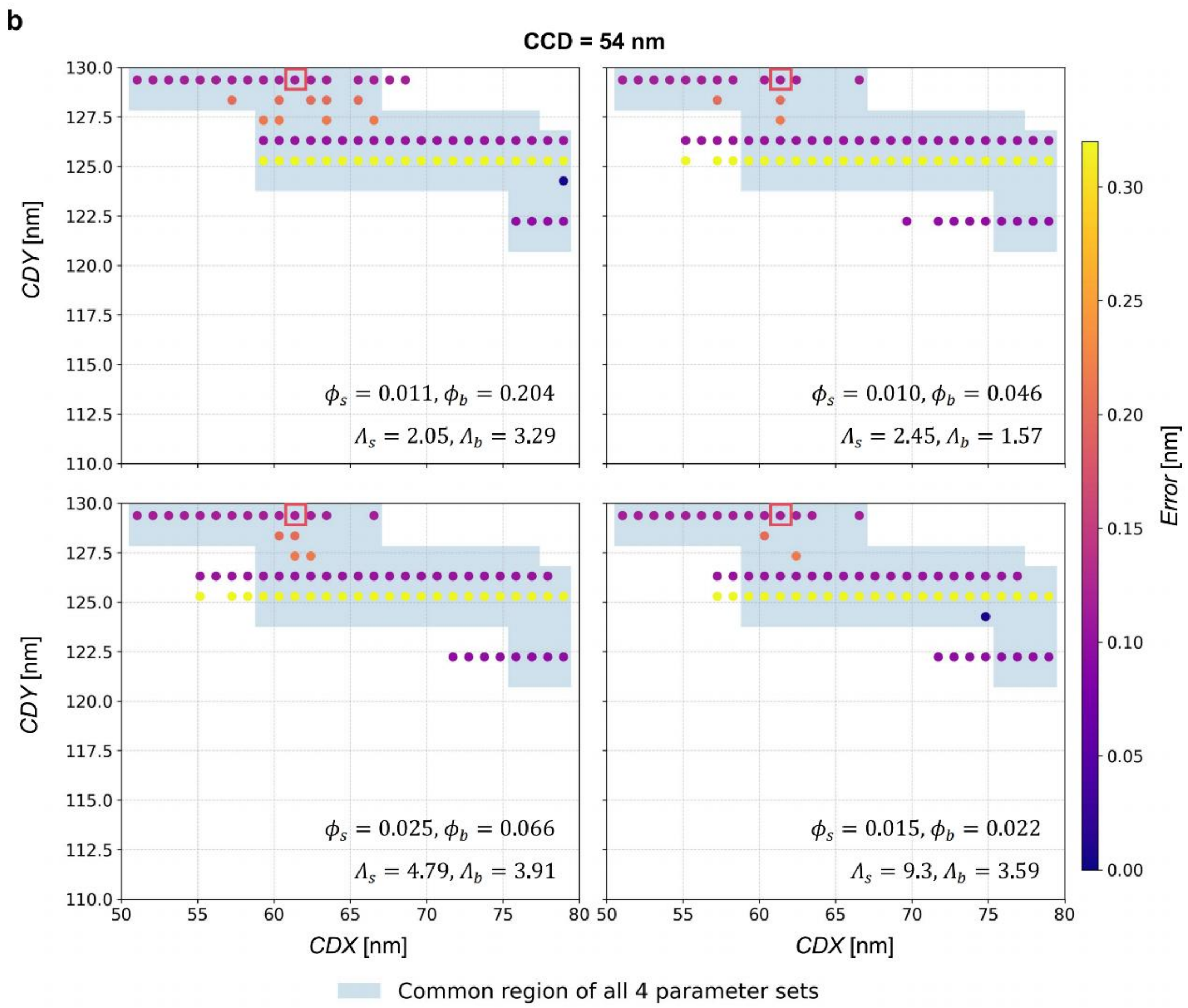
b
CCD = 54 nm
CDY [nm]
CDX [nm]
Error [nm]
$\phi_s = 0.011, \phi_b = 0.204$
$\Lambda_s = 2.05, \Lambda_b = 3.29$
$\phi_s = 0.010, \phi_b = 0.046$
$\Lambda_s = 2.45, \Lambda_b = 1.57$
$\phi_s = 0.025, \phi_b = 0.066$
$\Lambda_s = 4.79, \Lambda_b = 3.91$
$\phi_s = 0.015, \phi_b = 0.022$
$\Lambda_s = 9.3, \Lambda_b = 3.59$
Common region of all 4 parameter sets

Figure S16. Robustness analysis of the inverse-design process windows under different low-error calibrated parameter sets. (a) Feasible template regions for CCD = 52 ± 1 nm. (b) Feasible template regions for CCD = 54 ± 1 nm. The light-blue shaded regions denote the intersection of the feasible regions obtained from all four parameter sets. Red squares indicate experimentally validated template geometries.

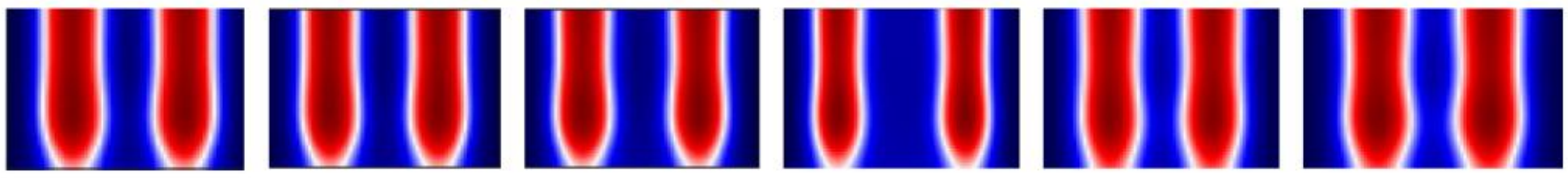

Figure S17. Cross-sectional structures of the DSA domains for peanut-shaped templates. Cases 1-6 correspond to the six peanut-shaped templates listed in Table 2.

9. Key simulation, optimization, and CNN training settings

For reference, the key settings used for the 3D OK simulations, BO, alternative surrogate models, and CNN training are summarized in **Tables S5-S8**.

Table S5. Key numerical settings used for the 3D OK simulations.

Setting	Value
PMMA volume fraction, f	0.28
Initial-field random seed	42
Grid spacing	0.2 Rg
Time step	$2.2 \times 10^{-5}\ t_0$
Number of iterations	1.6×10^5
Simulation height	$2L_0$

Table S6. Key settings used for RF-based BO.

Setting	Value
Initial samples	2 Sobol samples + 2 empirical seed samples
Initial-sample parameter order	$(\phi_s, \phi_b, \Lambda_s, \Lambda_b)$
Sobol sample 1	(0.0514, 0.3283, 0.6585, 2.9158)
Sobol sample 2	(0.0308, 0.1421, 3.3549, 0.3006)
Empirical seed sample 1	(0.17, 0.40, 10, 2)
Empirical seed sample 2	(0.20, 0.30, 8, 1)
Parameter bounds	ϕ_s, ϕ_b ∈ [0, 0.5]; Λ_s, Λ_b ∈ [0, 10], progressively refined during optimization
Surrogate model	Random Forest
RF settings	Number of trees = 100<br>Minimum samples per leaf = 1

Setting	Value
	Features considered at each split = 1
Acquisition function	Expected Improvement (ζ = 0.01)
EI candidate pool	5000 randomly generated candidate points
Batch size	1
Invalid-sample penalty	1000
CNN threshold	Classification threshold > 0.5 classified as perfect
Maximum evaluations	100
Stopping strategy	Optimization was terminated after reaching a stable low-error regime
Random seed	40

Table S7. Key settings used for alternative GP surrogate-model.

GP setting	GP-ARD	GP-isotropic
Kernel	1.0 × Matérn-5/2	1.0 × Matérn-5/2
Initial length scale	[1, 1, 1, 1]	1.0
Length-scale bounds	[10^{-2}, 10^{2}]	[10^{-2}, 10^{2}]
Length-scale treatment	Independent for $(\phi_s, \phi_b, \Lambda_s, \Lambda_b)$	Shared by all four variables
Length-scale optimization	Maximization of the log marginal likelihood	Maximization of the log marginal likelihood
Regularization, α	10^{-8}	10^{-8}
Optimizer restarts	2	2
Output normalization	Yes	Yes

Except for the surrogate-model specification, the initialization strategy, parameter bounds, acquisition function, candidate-pool size, batch size, penalty strategy, stopping criterion, and random seed were kept identical to those used in the RF-based BO.

Table S8. Key training settings used for the CNN classifier.

Setting	Value
Dataset split	Training / validation / test = 0.8 / 0.1 / 0.1
Data-splitting seed	42
Input	Single-channel, 128 × 128
Optimizer	Adam
Learning rate	0.001
Weight decay	0.0001
Batch size	64
Maximum epochs	20
Early stopping	Based on validation loss
Loss function	Class-weighted binary cross-entropy
Classification	Sigmoid output with validation-determined threshold = 0.73

Setting	Value
Training seed	Not fixed

References

S1. T. Ohta and K. Kawasaki, "Equilibrium Morphology of Block Copolymer Melts," *Macromolecules* 19, no. 10 (1986): 2621-2632. doi: 10.1021/ma00164a028.

S2. K. Yoshimoto, K. Fukawatase, M. Ohshima, et al., "Optimization of Directed Self-Assembly Hole Shrink Process with Simplified Model," *Journal of Micro/Nanolithography, MEMS, and MOEMS* 13, no. 3 (2014): 031305. doi: 10.1117/1.JMM.13.3.031305.

S3. S. Puri and Y. Oono, "Study of Phase-Separation Dynamics by Use of Cell Dynamical Systems. II. Two-Dimensional Demonstrations," *Physical Review A* 38, no. 3 (1988): 1542-1565. doi: 10.1103/PhysRevA.38.1542.

S4. M. Bahiana and Y. Oono, "Cell Dynamical System Approach to Block Copolymers," *Physical Review A* 41, no. 12 (1990): 6763-6771. doi: 10.1103/PhysRevA.41.6763.

S5. R. Garnett, *Bayesian Optimization* (Cambridge: Cambridge University Press, 2023). doi: 10.1017/9781108348973.

S6. C. Shang, J. Luo, W. Zeng, Y. Zhang, and S. Xiong, "Fast Simulation of Directed Self-Assembly for Contact Hole Multiplication with an Optimized Compact Model," in *Ninth International Workshop on Advanced Patterning Solutions (IWAPS 2025)*, *Proceedings of SPIE*, vol. 13991 (2025): 1399112. doi: 10.1117/12.3093234.

S7. L. Breiman, "Random Forests," *Machine Learning* 45, no. 1 (2001): 5-32. https://doi.org/10.1023/A:1010933404324.

S8. F. Hutter, H. H. Hoos, and K. Leyton-Brown, "Sequential Model-Based Optimization for General Algorithm Configuration," in *Learning and Intelligent Optimization*, *Lecture Notes in Computer Science*, vol. 6683 (2011): 507-523. doi: 10.1007/978-3-642-25566-3_40.

S9. D. Zhan and H. Xing, "Expected Improvement for Expensive Optimization: A Review," *Journal of Global Optimization* 78, no. 3 (2020): 507-544. doi: 10.1007/s10898-020-00923-x.

S10. A. Krizhevsky, I. Sutskever, and G. E. Hinton, "ImageNet Classification with Deep Convolutional Neural Networks," *Communications of the ACM* 60, no. 6 (2017): 84-90. doi: 10.1145/3065386.
